# A revised model of nuclear actin import: Importin 9 competes with cofilin, profilin, and RanGTP for actin binding

**DOI:** 10.1016/j.jbc.2025.111123

**Published:** 2025-12-30

**Authors:** Amanda J. Keplinger, Prithi A. Srinivasan, Sarah M. Christensen, Cristian Suarez, Alexander J. Ruthenburg

**Affiliations:** 1 Department of Molecular Genetics and Cell Biology, University of Chicago, Chicago, Illinois, USA; 2 Department of Biochemistry and Molecular Biophysics, University of Chicago, Chicago, Illinois, USA

**Keywords:** actin, cofilin, G-actin, kinetics, nuclear translocation, nuclear transport, protein import, protein–protein interaction, surface plasmon resonance, thermodynamics

## Abstract

While predominantly studied in cytoplasmic contexts, actin plays critical roles in the nucleus, regulating genome accessibility, transcription, and DNA repair. Cell-based studies have contributed to a widely accepted model in which the import factor importin 9 (IPO9) acts in concert with the actin filament–severing protein cofilin to transport actin into the nucleus. The classical nuclear localization signal on cofilin is thought to anchor IPO9 to cofilin-bound actin monomers, driving the formation of an import-competent tripartite actin–cofilin–IPO9 complex. Contrary to this established model of actin import, we demonstrate that IPO9 directly binds to monomeric actin with midnanomolar affinity and, rather than promoting IPO9–actin complex formation, cofilin competitively inhibits the binding of IPO9 to actin. To define the mechanism of IPO9 binding, we subject monomeric actin to competitive IPO9 binding in the presence of well-established actin-binding molecules and find proteins that engage either the barbed face, profilin, or pointed face, DNase I, competitively limit IPO9–actin complex formation, whereas sterically less demanding binding partners, thymosin beta-4 and latrunculin B, do not. Consistent with these findings, we demonstrate that IPO9 modestly decreases the rate of actin filament assembly, a process that requires both actin faces, and that IPO9 exhibits minimal binding to actin filaments. Finally, we identify unexpected affinity between the nuclear import release factor RanGTP and monomeric actin; however, a tripartite IPO9–actin–RanGTP complex does not form. The competitive interactions observed between IPO9 and cytoplasmic actin-binding proteins suggest that dynamically coupled equilibria mediate the nuclear transport of actin monomers.

Actin is an extremely abundant cytoskeletal protein that plays fundamental roles in key processes, including cell motility, cytokinesis, and the maintenance of cell shape and polarity. Central to these functions is the dynamic equilibrium between actin polymer states, monomeric G-actin *versus* polymeric F-actin, mediated by a range of cytoplasmic actin-binding proteins (ABPs) (1, 2). Members of the actin-depolymerizing factor family, including cofilin, serve as the major regulators of actin filament breaking in the cell, severing filaments to contribute to the sizeable cytoplasmic pool of monomeric actin (3, 4, 5). This monomeric actin pool is further maintained through selective binding by profilin and thymosin beta-4. While profilin-bound actin can ultimately contribute to filament assembly (6, 7), thymosin beta-4 strongly sequesters these actin monomers, preventing them from being incorporated into a growing filament (8, 9). This equilibrium of exchange between profilin and thymosin beta-4 ensures the existence of a storage pool of actin monomers and serves to regulate their contribution to filament formation in the cytoplasm (10).

In addition to this dynamic cytoplasmic pool, the nuclear pool of actin is essential for maintaining proper nuclear functions and cellular homeostasis (11, 12, 13). Nuclear monomeric actin is a critical component of chromatin-modifying complexes, such as BAF and INO80 (14), and promotes both transcriptional initiation and elongation by RNA polymerases (15, 16, 17, 18). Nuclear actin filaments have been implicated in long-range chromatin movements (19, 20, 21) and are known to form scaffolds to support the repair of DNA double-stranded breaks (11, 12). Dysregulation of nuclear actin abundance causes aberrant gene regulation and is associated with cancer, cardiovascular disease, and neurodegeneration phenotypes (13, 22, 23, 24, 25, 26, 27, 28). Notably, the nuclear and cytoplasmic actin pools are directly linked—large increases or decreases in the availability of monomeric actin in the cytoplasm, through tissue repair, small-molecule actin polymerization inhibitors, and disease states, have been shown to alter the nucleoplasmic pool of actin and disrupt nuclear actin activities (25, 29, 30). It follows that sophisticated cellular mechanisms are required to maintain the balance between the cytoplasmic and nuclear pools of actin.

Transport of proteins across the nuclear membrane requires passage through hydrophobic nuclear pores. A class of large, flexible chaperone proteins—composed of importins, exportins, and biportins—use their charged inner surface to mediate specific binding to protein cargo, whereas their hydrophobic outer surface favorably interacts with the nuclear pore. The mechanism of nuclear actin export has been well defined: exportin-6, a mammalian export chaperone protein, recognizes nuclear monomeric actin bound by profilin, profilin–actin, for transport back into the cytoplasm (31). Importantly, through this nuclear export pathway, the concentration of actin within the nucleus is directly coupled with that of profilin, contributing to the regulation of polymer states across both cellular compartments.

In contrast to the detailed mechanistic information available regarding actin’s nuclear export (31, 32, 33), less is known about how nuclear import machinery responds to the dynamic actin pool in the cytoplasm to facilitate import. An RNAi screen of known nuclear import factors identified the chaperone protein importin 9 (IPO9) as the primary factor responsible for the nuclear import of actin (34). Importins canonically recognize and bind their cargos through defined lysine-rich motifs known as classical nuclear localization signals (cNLSs) (35). However, actin lacks a cNLS, suggesting that it may not directly bind to IPO9 for nuclear transport. Analogous to actin’s nuclear export with profilin, it is thought that actin is brought into the nucleus in complex with the ABP cofilin (CFL1). Cofilin has a canonical bipartite cNLS, which remains accessible when cofilin is bound to actin monomers (33, 36, 37). This cNLS has been proposed to recruit IPO9 to cofilin-bound actin monomers, resulting in the formation of a tripartite actin–cofilin–IPO9 import complex (Fig. 1*A*) (34, 38, 39). In support of this model, siRNA knockdown of cofilin slightly decreases the nuclear localization of the preferentially monomeric actin mutant R62D (34). Furthermore, perturbing actin polymerization through latrunculin B (LatB) treatment drastically increases nuclear concentrations of both actin and cofilin, implying a mechanism in which actin and cofilin are concomitantly brought into the nucleus (36). However, IPO9 can bind cargo independently of a cNLS, so it remains to be determined whether the cofilin cNLS is necessary to mediate IPO9–actin binding (40, 41, 42). The evidence supporting this prevalent model (23, 24, 43, 44, 45, 46) is largely indirect; the precise dynamics of the binding affinities between IPO9, actin, and cofilin have not been directly tested *in vitro*.

**Figure 1:**
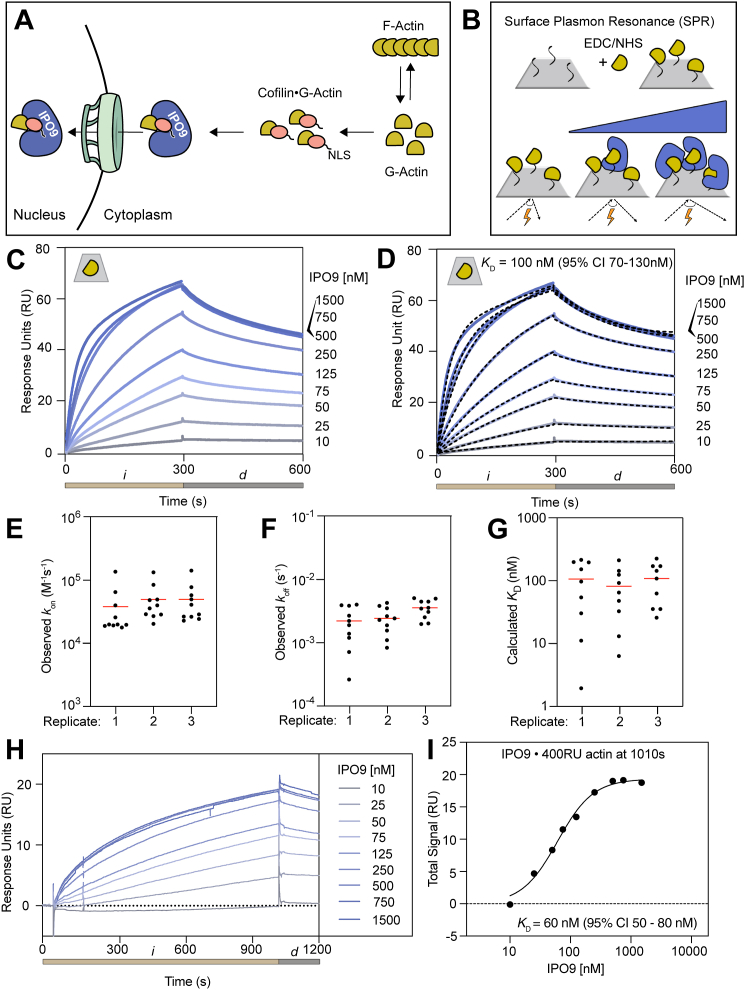
**IPO9 binds monomeric actin with nanomolar affinity**. *A*, cartoon depicting the established model of actin nuclear import, wherein the classical nuclear localization signal (cNLS) of cofilin (*peach*) anchors actin (*yellow*) to IPO9 (*blue*), resulting in the formation of a tripartite cofilin–actin–IPO9 import-competent complex. *B*, schematic of a surface plasmon resonance (SPR) experiment where actin (*yellow*) is covalently linked to the CM5 sensor chip (*gray surface*) through EDC carbodiimide linkage. Afterward, varying concentrations of analyte (IPO9, *blue*) are flowed over the sensor chip in a direct binding assay where analyte binding changes the angle of reflection measured (reported as response unit [RU] change). *C*, representative sensorgram plot for IPO9 affinity measurement. Binding curves for each concentration of IPO9 from 0 to 1500 nM are shades of *blue* with concentration denoted on the *right*. Change in RUs is proportional to the analyte protein bound. *Tan bar* (*i*) corresponds to 300 s injection of IPO9, and *gray bar* (*d*) corresponds to 300 s dissociation phase, where buffer is applied at the same flow rate (30 μl/min). *Gray* trapezoid with *yellow* actin monomer in the *upper left* denotes actin monomer is covalently linked to the CM5 chip. *D*, representative kinetic fit (*black dashes*) overlaid on raw sensorgram curves shown in (*C*) for each concentration using a two-state binding model with local fitting in Biacore8k software. Kinetic fit data for each concentration point in each replicate are displayed. *E*, observed *k*_on_ and (*F*) observed *k*_off_ for kinetic fits and (*G*) resultant *K*_*D*_ calculated for each concentration for each replicate. *Horizontal line* represents the arithmetic mean for each fit. *H*, sensorgram plot for elongated injection (1020 s, 10 μl/min) to approach equilibrium. *i*, analyte injection; EDC, 1-ethyl-3-(3-dimethylaminopropyl) carbodiimide hydrochloride; IPO9, importin 9.

Here, we used surface plasmon resonance (SPR) and actin polymerization assays to probe the mechanism by which IPO9 binds to cytoplasmic actin for nuclear transport. In contrast to the cofilin-dependent model of actin’s nuclear import, we identify that IPO9 has specific affinity for actin monomers in the midnanomolar range and observe that this binding is directly competitive with cofilin–actin binding. Binding to actin by the ABP profilin is similarly competitive, potentially indicating an overlapping surface on the barbed face of actin for binding by IPO9. In further probing the pointed face of actin monomers through competitive binding assays with DNase I, we find a stark decrease in actin–IPO9 binding when the actin-pointed face is engaged by DNase I. Finally, we find that actin binds directly to the nuclear import release factor, RanGTP, but that this binding is not concomitant with actin–IPO9 binding. Reconciling our data with previous literature, we posit that the observed dependence of nuclear actin transport on cofilin in cell-based assays is a result of filament disassembly processes upstream of the actin–IPO9 binding interaction instead of a requirement for cofilin itself in the nuclear transport step. These findings merit a re-evaluation of prior *in cellulo* work that considers the primary role of ABPs like cofilin in regulating the availability of import-competent actin monomers in the cytoplasm.

## Results

### Importin 9 can directly bind to monomeric actin

Given the dearth of *in vitro* binding data characterizing the interactions between IPO9, actin, and cofilin, we sought to determine whether IPO9 alone has affinity for actin with purified proteins (Fig. S1*A*). In an initial pulldown with glutathione-*S*-transferase (GST)-IPO9, we observed specific direct binding between actin and IPO9 in the absence of cofilin (Fig. S1*B*). Unfortunately, because of nonspecific binding of actin to glutathione beads, subsequent pulldowns were inconsistent and difficult to quantify relative to background actin-bead binding. As monomeric actin is thought to be preferentially imported into the nucleus (9), an additional complication of this assay format was the requirement for actin concentrations near the critical concentration of filament formation (0.5 μM), making it difficult to control for polymerization state (47). To circumvent these challenges and more quantitatively evaluate the specific binding affinity between IPO9 and monomeric actin, we employed SPR experiments.

With a concentration regime of actin below the critical concentration of filament formation (20 nM), we tethered ATP–actin monomers to the chip surface through activated ester crosslinking (Fig. 1*B*). In this method, the ligand is nonspecifically coupled to the surface in many orientations, which is critical for a large chaperone protein like IPO9 that tends to be sterically constrained as it wraps around its cargo (35, 48). This experimental design has previously been used to characterize monomeric actin binding partners (49). Using a concentration series of IPO9 (10–1500 nM), we observed a specific binding signal approaching saturation with increasing concentrations of IPO9 in each injection (*i*), followed by characteristic dissociation upon buffer flow (*d*) (Figs. 1*C*, S1*C*). Applying a two-state kinetic fit model, we find good accordance among three replicates for *k*_on_ and *k*_off_ values across the concentration series over three replicates (Fig. 1, *D* and *E*, *F*). The *k*_off_ rate constant measurements (Fig. 1*F*) imply a half-life of IPO9–actin complexes in the hundreds-to-thousands of seconds, which would be amenable to the timescale of nuclear import (50, 51). From these measurements, we calculated the arithmetic mean *K*_*D*_ to be 100 nM (95% confidence interval [95% CI], 70–130 nM) (Fig. 1*G*). We repeated these binding assays with a longer IPO9 injection period to approach equilibrium at the end point of the injection. Applying a Hill equilibrium fitting model to these data, we find an affinity of 60 nM (95% CI, 50–80 nM) (Figs. 1, *H* and *I*, S1, *D*–*E*). The accordance of the two fitting models gives us confidence in the accuracy of the measurement. An affinity between IPO9 and actin of 60 to 100 nM suggests that this binding is likely to be physiologically significant, as it lies below the cellular concentrations of actin and IPO9 of ∼13.2 μM and ∼0.5 μM, respectively (52, 53). It is on the tighter end of the range of direct binding affinities of actin and other ABPs (100 nM–10 μM) and in the regime of IPO9 for its other characterized cargo (1 nM–1 μM) (35, 42, 48, 52). These data argue that direct actin–IPO9 binding equilibrium and consequent import of the complex could plausibly be coupled to the cytosolic G-actin pools.

### IPO9 limits actin filamentation through profilin-like binding

**Figure 2:**
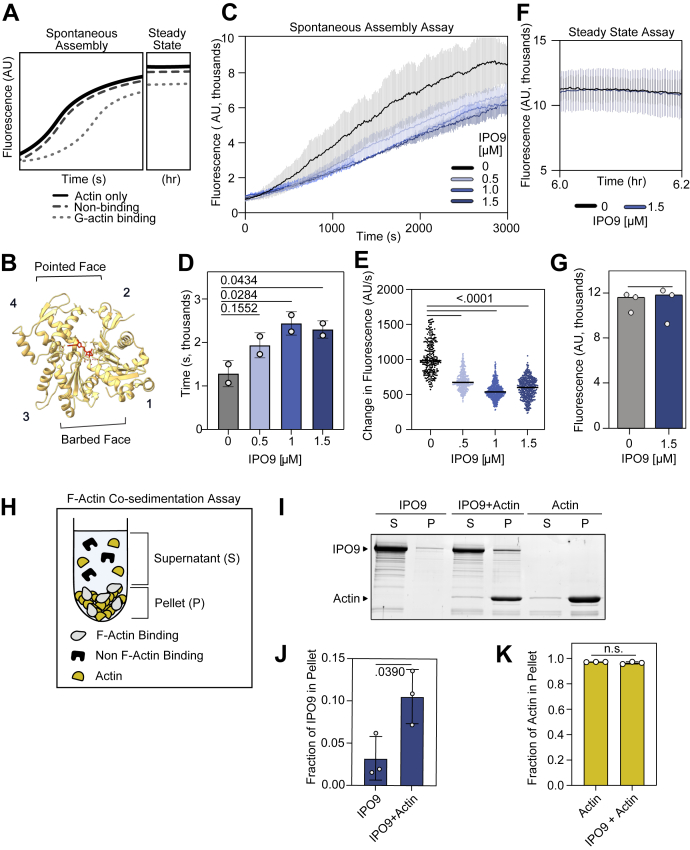
**IPO9 binds the barbed face of actin but does not strongly sequester**. *A*, schematic depicting potential kinetic time-course data of an actin polymerization assay for the spontaneous assembly (s) and steady-state (hr) timescales. Possible outcomes displayed for actin alone (*black*), nonsequestering (*dark gray*), and sequestering (*light gray*) ABPs in pyrene assembly assays. *B*, *cartoon* depicting subdomains 1 to 4 of a single actin monomer (1). The barbed face surface corresponds to subdomains 1 and 3, whereas the pointed face corresponds to subdomains 2 and 4. In the *middle*, there is the nucleotide-binding cleft (*red*). Barbed face binding will result in decreased actin filament assembly rate (Protein Data Bank code: 3HBT) (54). *C*, IPO9 decreases the rate of actin filament assembly in the pyrene-labeled actin spontaneous assembly assay. About 20% pyrene-labeled actin (1.5 μM) in the presence of (0 μM) (*black*), (0.5 μM) (*light blue*), (1.0 μM) (*medium blue*), and (1.5 μM) (*dark blue*) IPO9, with standard deviation bars indicated for each curve. *D*, time each spontaneous assembly reaction condition takes to go from *t*_.1∗max_ to *t*_.5∗max_. Maximum AU signal calculated from steady-state actin-alone reaction (n = 2, ordinary one-way ANOVA with Dunnett's correction for multiple comparisons, *p* values for each comparison are indicated above the bar). *E*, plot of a sliding 300 s window of slopes measured from *t*_.1∗max_ to *t*_.5∗max_ (ordinary one-way ANOVA with Dunnett's correction for multiple comparisons). *F*, IPO9 is not a strong actin-sequestering ABP. Steady-state pyrene–actin assembly assay, with actin (1.5 μM) and either control (no IPO9, *black*) and experimental (+IPO9, *blue* [1.5 μM]) conditions after preincubation for 6 h. *G*, IPO9 does not alter the total fluorescence signal in the steady-state filament assembly assay. Average fluorescence intensity (AU) of steady-state pyrene polymerization assay with and without IPO9. Quantification of total absorbance at 6.05 h (ns = not significant in a two-tailed Welch’s *t* test, *p* = 0.9062, n = 3). *H*, schematic of F-actin cosedimentation assay following ultracentrifugation (25). *I*, representative 15% SDS-PAGE after ultracentrifugation of actin (3 μM) (42 kDa), IPO9 (3 μM) (115 kDa), or actin + IPO9 (3 μM, 3 μM). P, pellet; S, supernatant. Shift of IPO9 into the P fraction in the presence of actin would indicate F-actin binding. *J*, IPO9 quantification shows similar amounts of protein present in pellet fraction relative to total pellet + supernatant signal between IPO9 alone and IPO9 + actin conditions (n = 3). *p* Value indicated from two-tailed Welch’s *t* test. *K*, quantification of actin signal present in pellet fraction for actin-only sample *versus* actin + IPO9 sample. *p* Value indicated from two-tailed Welch’s *t* test is not significantly different (*p* = 0.3007). ABP, actin-binding protein; IPO9, importin 9.

To better understand the mode by which IPO9 binds actin monomers and to further probe its capacity to interact with filaments, we used pyrene–actin assembly and filament cosedimentation assays (Fig. 2*A*). G-actin sequestering proteins like profilin and thymosin beta-4 act by reducing the effective concentration of free monomeric actin through binding the barbed face of actin (Fig. 2*B*, subdomains [SDs] 1 and/or 3), thus diminishing the rate of filament assembly (10, 55, 56). Similarly, pointed-face binding proteins like DNase I are capable of enhancing the rate of actin filament turnover, also contributing to decreased filament assembly over time (57, 58). Strikingly, we observed a significantly increased time of spontaneous filament assembly from *t*_10%_ to *t*_50%_ and a reduced rate of filament assembly in the presence of increasing concentrations of IPO9, suggesting that IPO9 is *also* capable of binding and mildly sequestering monomeric actin (Figs. 2, *C* and *D*, S2, *A*–*B*). Given the directionality of actin filament formation, in which the pointed face of one actin monomer binds the barbed face of an elongating filament, the diminished filament assembly we observe implies that IPO9 engages and partially occludes one or both faces of actin (10, 55, 59). We further probed the stability and extent of actin monomer sequestration by IPO9 *via* a steady-state pyrene–actin assembly assay and found that IPO9 is unable to disrupt existing actin filaments or sequester monomers over long timescales (Fig. 2, *E* and *F*). This behavior differs from the strong sequestration activity of thymosin beta-4 but is comparable, though weaker, to that of profilin (7, 8, 10).

Having observed this specific affinity of IPO9 for monomeric actin, we further sought to examine whether IPO9 also displayed affinity for actin filaments with actin cosedimentation assays (Fig. 2*G*) (55). Compared with an IPO9-only control, we observed only a slight increase of sedimented IPO9 in the presence of F-actin (Figs. 2, *H* and *I*, S2*A*), indicative of weak binding to filamentous actin relative to the high affinity for G-actin we observe by SPR. Similarly, in a filament-promoting buffer environment, IPO9 is unable to shift the equilibrium of actin filaments toward monomers relative to an actin-only control, in contrast to a strong sequestering protein like thymosin beta-4 (Fig. 2*J*). Collectively, these data argue that monomeric actin is preferentially bound by IPO9 in a manner that limits the accessibility of one or both filament-forming faces, a conclusion that aligns with the speculated preferential nuclear import of actin monomers (36).

### IPO9 and cofilin competitively bind to actin monomers

**Figure 3:**
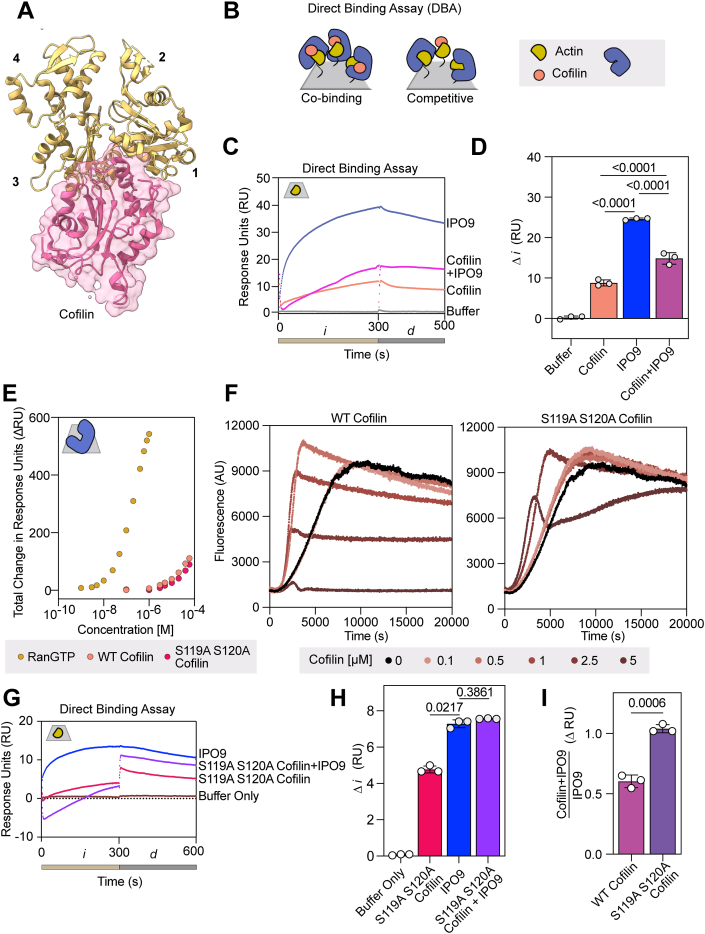
**Cofilin and IPO9 do not cobind actin**. *A*, structural depiction of cofilin (*pink*) binding to the barbed face of actin (adapted from ATP–actin structure (Protein Data Bank code: 8OH4 [*yellow*] (61)). A structure of cofilin–monomeric actin does not exist; therefore, we have adapted the above structure of cofilin bound to an actin filament. Cofilin has been shown to bind multiple faces of actin, and this is one possible orientation. *B*, schematic depicting outcomes of coinjection of saturating concentrations of IPO9 (*blue*) and cofilin (*peach*) in a direct binding assay with immobilized actin (*yellow*) on an SPR chip (*gray*); these two scenarios represent either competitive or cooperative/cobinding for IPO9 and cofilin with actin. *C*, representative sensorgram of direct binding assay with buffer-only control, IPO9 alone, cofilin alone, and IPO9 + cofilin coinjection conditions. *Tan bar* corresponds to the injection phase, *i*, and *gray bar* depicts *d* dissociation phase. *D*, quantification of change in signal (dRU) between 10 and 290 s (*p* values computed by one-sided repeated-measures ANOVA with Tukey's correction for multiple comparisons between means of change in RU indicated by bars, n = 3). *E*, SPR with IPO9 crosslinked to the chip and RanGTP (positive control for IPO9 binding, *mustard*) (0–1 μM) and cofilin, WT (*peach*), and actin binding mutant S119A + S120A (*pink*) (0–55 μM) were flowed over to determine affinity to IPO9. *F*, pyrene–actin polymerization assays with increasing concentrations of WT or S119A S120A mutant cofilin with indicated concentrations. Shift of initial rate of signal increase to the *left* with a steeper slope indicates the ability of cofilin to sever filaments, followed by approach to steady state at a lower overall fluorescence, indicates monomer chaperone activity. *G*, representative sensorgram of direct binding assays with mutant S119A S120A cofilin (*pink*), IPO9 (*blue*), or the combination of both (*purple*). *H*, mutant S119A S120A cofilin does not significantly decrease IPO9 binding signal when coinjected. Quantification of change in signal (DRU) between 10 and 290 s *p* values computed by one-sided repeated-measures ANOVA with Tukey's correction for multiple comparisons between means of change in RU indicated by bars (n = 3). *I*, quantification of the signal of cofilin + IPO9 condition over the IPO9-only signal for WT or mutant cofilin. *p* Values indicated were computed by Welch’s two-sided *t* test. IPO9, importin 9; SPR, surface plasmon resonance.

The direct high-affinity binding between IPO9 and actin that we have established eliminates the necessity of cofilin for IPO9–actin binding, raising the question of whether cofilin plays any role in modulating this interaction as proposed (34). Intriguingly, one orientation that cofilin binds partially occludes the actin-barbed face (60) indicating that steric hindrance could limit concomitant barbed-face actin binding by both cofilin and IPO9 (Fig. 3*A*). In a pulldown with GST-IPO9 and actin, we saw that direct binding between IPO9 and actin decreased with the addition of cofilin, suggesting IPO9 and cofilin may compete for actin rather than binding cooperatively (Fig. S1*B*). We used SPR to robustly probe whether the presence of cofilin impacts the affinity of IPO9 for actin, where the size disparity between cofilin and IPO9 (18 kDa *versus* 115 kDa) enables clear attribution of respective binding. In this format, we measure the cofilin–G-actin affinity to be *K*_*D*_ = 20 ± 7 μM (Fig. S3, *A* and *B*), which is within the range of affinities others have measured for cofilin (62, 63).

Next, we employed a direct binding assay in which we coinjected premixed IPO9 (3 μM) and cofilin (55 μM) over immobilized actin (Fig. 3*B*). If cofilin could enhance actin–IPO9 binding as previously suggested, this premixed condition would be expected to produce a higher signal relative to IPO9 or cofilin alone (64). Instead, we observed *decreased* signal relative to IPO9 alone and increased signal relative to cofilin alone (Figs. 3, *C* and *D*, S3*C*). This was consistent with the immobilized actin binding either cofilin or IPO9, but not both proteins concurrently, suggesting cofilin and IPO9 compete for an overlapping interface on actin monomers. Such apparent competition could also be observed if IPO9 was binding cofilin and being competed off actin, as this would decrease the effective concentration of both proteins, which could bind the actin on the chip. To rule out this possibility, we separately immobilized IPO9 to the chip using activated ester crosslinking and examined its ability to bind cofilin. After validating that the immobilized IPO9 could bind its canonical cargo release factor RanGTP in the expected low-nanomolar affinity regime, we employed a concentration series of cofilin (0–60 μM) and observed only minimal binding to IPO9 at the highest concentration points (Fig. 3*E*) (65). We were unable to use an equilibrium fit model for this interaction as it does not reach saturation, but kinetic fit models predicted a *K*_*D*_ in the millimolar range (data not shown). This suggests that cofilin does not bind IPO9 tightly enough to significantly diminish the pool of IPO9 available for actin binding. As such, these results confirm that the apparent competition observed in the direct binding assay is between IPO9 and cofilin for the same population of immobilized actin monomers.

As an alternative means of examining the nature of the competition observed in direct binding assays, we employed a cofilin double point mutant, S119A S120A cofilin. While this mutant has a comparable affinity for IPO9 when compared with WT cofilin (Fig. 3*E*), it has reduced ability to bind actin (5, 66, 67). We confirmed the reduced actin binding activity of this cofilin mutant through a pyrene–actin spontaneous assembly assay; in the WT cofilin case, we see steeper initial filament formation because of filament-severing activity creating nucleation sites, and this effect is muted with the mutant (Fig. 3*F*). In the direct binding assay, while we observed moderate binding of this mutant cofilin to actin at high concentrations (55 μM), the premixed combination of IPO9 and mutant cofilin produced a statistically indistinguishable change in signal comparable to that of IPO9 alone, indicating that the mutant is unable to effectively compete with IPO9 for binding actin because of its reduced affinity for actin (Figs. 3, *G* and *H*, S3*D*). As compared with WT cofilin, the proportion of signal of IPO9 plus S119A S120A cofilin relative to IPO9 alone is greatly increased (Fig. 3*I*). These results confirm that the reduced IPO9–actin complex formation in the presence of cofilin results from cofilin–actin binding rather than cofilin–IPO9 binding, and that WT cofilin and IPO9 compete to bind a shared population of monomers—a consequence that may arise either from an overlapping actin-binding interface or negative allosteric perturbations to the structure of the actin monomer.

We also probed for potential cooperative binding between cofilin and IPO9 in an orthogonal assay commonly used in SPR, a surface competition A–B assay (68, 69). In this format, the first protein injection of cofilin (55 μM) saturates the actin-bound chip (*i)*, followed by a secondary protein injection (*i’*) of IPO9 (3 μM), and the relative binding is compared with a buffer-only *i* condition (Fig. S4*A*). After saturation of actin with cofilin, we do not observe a greater IPO9 signal in the cofilin-then-IPO9 condition (*purple*) compared with the buffer-then-IPO9 condition (*blue*), confirming that cofilin does not promote the ability of IPO9 to bind to actin. Together, these three different experimental approaches all support the interpretation that IPO9 and cofilin compete for actin monomers–a finding incongruous with the conventional model of cofilin–actin import through a single IPO9 binding event.

### Competitive binding assays to characterize the IPO9–actin binding mechanism

**Figure 4:**
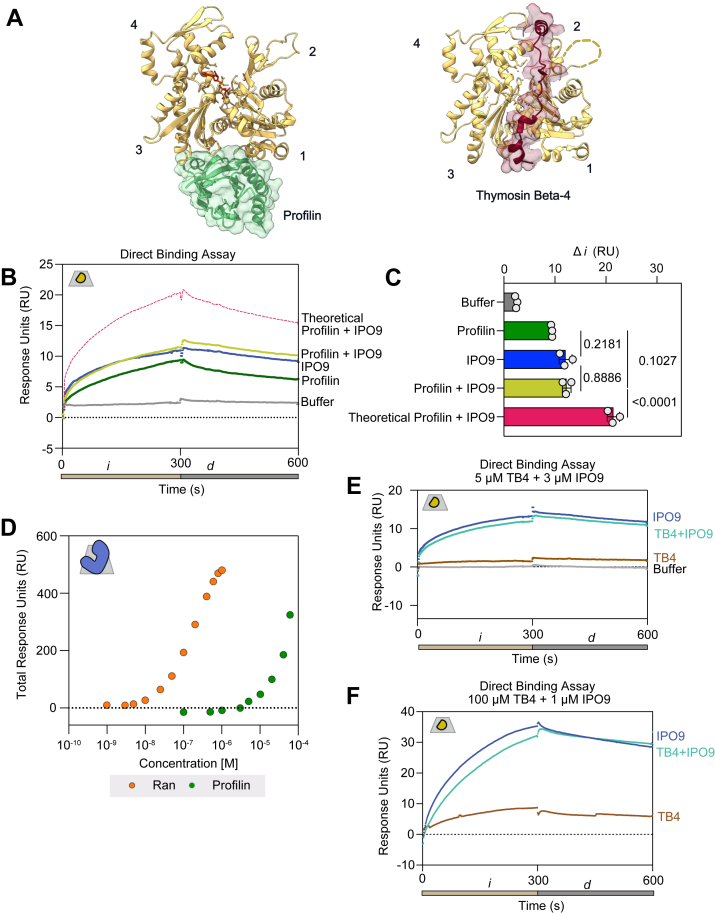
**IPO9 competes with profilin for actin monomer binding**. *A*, structural representation of profilin (Protein Data Bank [PDB] code: 2BTF) and thymosin beta-4 (PDB code: 4PL7) binding to an actin monomer (PDB code: 1WNK) (10, 70, 71). *B*, direct binding assay comparing coinjection of IPO9 (3 μM) and profilin (25 μM). Sensorgram signal is presented as follows: profilin (*green*), IPO9 (*blue*), and buffer (*gray*); also contoured is the theoretical additive signal of IPO9 and profilin independently and simultaneously cobinding (*dashed pink*). *C*, quantification of change in resonse unit (RU) signal from 10 to 290 s for all replicates of the experiment in *B* with (*p* values from repeated-measures one-way ANOVA with Tukey's correction for multiple comparisons, n = 3). Signal is not additive, and there is not a significant difference between IPO9 and profilin signal. *D*, profilin has a very mild affinity for IPO9. Affinity curves showing total RU change for different concentrations of profilin (0–60 μM) binding to IPO9 covalently linked to the chip, compared with the positive control of RanGTP binding (0–1 μM). *E*, representative sensorgram of direct binding assay with IPO9 (3 μM) (*blue*) or thymosin beta-4 (5 μM) alone (*brown*) or the combination of the two proteins (*teal*). *F*, representative sensorgram of direct binding assay with IPO9 (3 μM) (*blue*) or thymosin beta-4 (100 μM) alone (*brown*) or the combination of the two proteins (*teal*). IPO9, importin 9.

Instead of IPO9 only being able to engage cofilin-bound actin as formerly postulated, our results suggest that any postfilament disassembly or storage form of cytoplasmic G-actin could be a potential substrate for nuclear import. Much of the monomeric actin present within the cytoplasm is maintained through contact with other ABPs (52). For example, thymosin beta-4 and profilin compete to chaperone the pool of ATP–actin monomers, with thymosin beta-4 strongly sequestering monomers and profilin having flexibility to exchange its bound monomers to other ABPs or elongating filaments (6, 7, 8, 10). In light of the dynamic exchange of monomeric actin between these actin-sequestering factors in the cytoplasm, and their engagement of an actin-barbed face region overlapping with the cofilin–actin binding surface (Fig. 4*A*), we wanted to determine whether profilin or thymosin beta-4 binding to actin could also promote or inhibit its competence for nuclear import by IPO9.

To begin, we employed direct binding assays by coinjecting preincubated IPO9 (3 μM) and profilin (25 μM) over immobilized actin. Despite the significant size disparity between profilin and IPO9 (14 kDa *versus* 115 kDa), profilin has a high affinity for ATP–G-actin (6) and is likely less sterically hindered in binding a larger proportion of actin on the chip relative to IPO9, which is thought to wrap around its cargo (48). Thus, we observed nearly indistinguishable signals for profilin alone, IPO9 alone, and premixed IPO9 and profilin (Fig. 4, *B* and *C*, Fig. S5, *A* and *B*). These data rule out the possibility of concomitant binding of monomeric actin by *both* profilin and IPO9, as well as profilin and IPO9 engaging entirely distinct pools of monomeric actin, strongly suggesting competitive binding for an overlapping actin surface. Critical to this interpretation, we also find that profilin does not strongly bind IPO9 (Fig. 4D), which implies that profilin, like cofilin, is capable of competing with IPO9 for actin binding.

We then used a similar assay to determine whether IPO9 could bind thymosin beta-4–sequestered actin monomers by flowing premixed IPO9 (3 μM) and thymosin beta-4 (5 μM) over immobilized actin. As thymosin beta-4 is a 5 kDa protein, we were unable to detect appreciable signal corresponding with its binding to actin relative to buffer alone. This is consistent with a previous work, in which over 10 times the quantity of bound ligand was required to generate a discernible analyte binding signal for thymosin beta-4 (72). Nonetheless, we observed no significant difference in signal between premixed IPO9 and thymosin beta-4 compared with IPO9 alone (Figs. 4*E*, S5, *C*–*D*), suggesting that at these concentrations, thymosin beta-4 is unable to prevent or promote IPO9 binding to actin. The concentration of thymosin beta-4 utilized in this direct binding assay is between 2- and 10-fold above its reported *K*_*D*_ for G-actin (10, 62); however, thymosin beta-4 has been shown to exist at up to 100-fold greater concentration in cells (73). To examine the competition between IPO9 and thymosin beta-4 in this higher concentration regime, we attempted a competition assay between IPO9 and 100 μM thymosin beta-4 (Fig. 4*F*). In this case, we do see a measurable signal from thymosin beta-4–actin binding alone, and a marginal decrease in the response unit (RU) signal when IPO9 and thymosin beta-4 are combined. This indicates that there is no apparent cobinding or competition with actin by both IPO9 and thymosin beta-4 in physiologically relevant concentration regimes.

**Figure 5:**
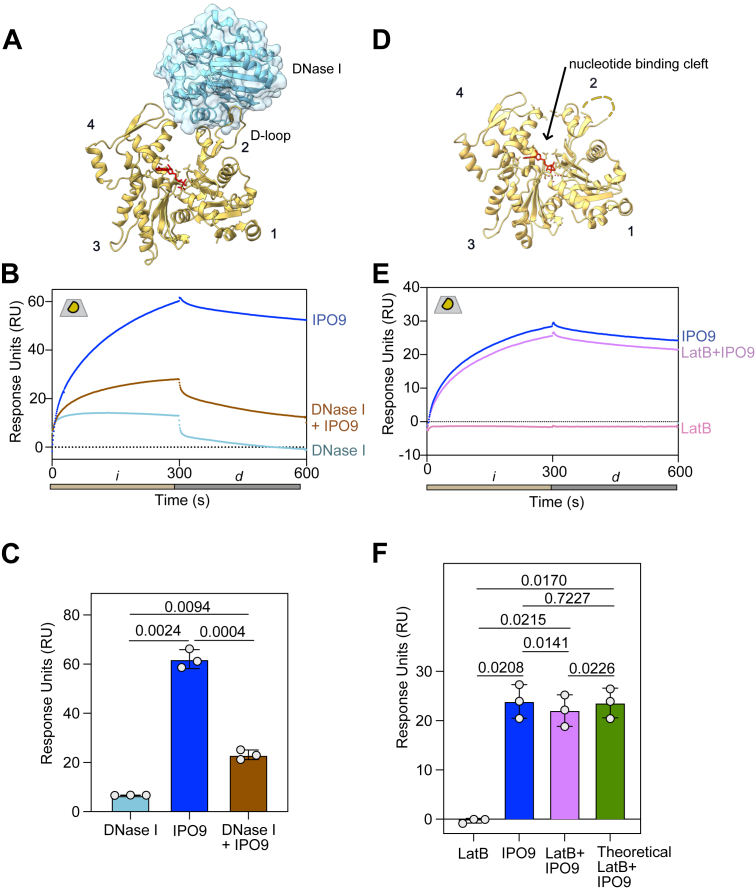
**DNase I and latrunculin B (LatB) compete for actin binding with IPO9**. *A*, structural representation of DNase I binding to actin (Protein Data Bank code: 1ATN) and actin alone (Protein Data Bank code: 3HBT) (54, 77). *B*, representative sensorgram of surface competition assays with between IPO9 (1 μM) and DNase I (5 μM). *C*, quantification of n = 3 replicates for surface competition assays. *D*, structural representation of the actin monomer pointing out the nucleotide-binding cleft, the region that the small molecule LatB binds to. *E*, surface competition assay with IPO9 (1 μM) and LatB (12.5 μM) (previously shown to bind actin with 50 nM affinity) (78). *F*, quantification of change in signal for surface competition assay. Repeated-measures one-way ANOVA with Tukey's correction for multiple comparison results displayed on a graph. IPO9, importin 9.

IPO9 and other importins are highly flexible and often wrap around their cargo to facilitate shielding for transport through the nuclear pore (74, 75, 76). Having implicated the actin barbed face in IPO9 binding, we sought to examine whether a distinct face of actin may also participate in IPO9 binding. DNase I is known to bind at the pointed face of actin, in a region known as the D-loop, with surprisingly robust affinity (Fig. 5*A*) (79). In a competition assay with 5 μM DNase I, we observe marked competition of IPO9 binding signal—the intermediate binding signal of the IPO9–DNase I mixture is a composite of the individual DNase I and IPO9 (Figs. 5, *B* and *C*, S6*A*). With much higher concentrations of DNase I and the same concentration of IPO9, the competition becomes DNase I dominated (Fig. S6*B*). These data are consistent with the hypothesis that IPO9 wraps around the actin monomer and contacts both the barbed and pointed faces of actin, although it is formally possible that negative allostery accounts for the mutually exclusive binding.

Finally, we wanted to investigate the importance of the actin nucleotide-binding cleft on IPO9 binding by using LatB, a small molecule that destabilizes actin filaments upon binding between SDs 2 and 4 (80). To probe whether binding of LatB inhibits binding of IPO9, we performed a surface competition assay with immobilized actin and found it is unable to inhibit IPO9 binding (Figs. 5, *C* and *D*, S6*D*). This indicates that neither specific binding to the nucleotide-binding cleft nor any actin conformational effect of LatB binding is critical for the binding of IPO9 to the actin monomer (81).

### IPO9–actin binding is diminished in the presence of the canonical release factor, RanGTP

Having examined actin recognition and binding by IPO9, we wanted to probe the mechanism by which actin may be released from IPO9 in the nucleus. For other importins, cargo release is generally thought to be mediated by the nuclear protein, RanGTP. Upon RanGTP binding to an importin–cargo complex, the cargo dissociates, leaving an importin–RanGTP complex, which then diffuses out of the nucleus (48). However, the release mechanism for IPO9 has been shown to have variable dependence on RanGTP binding (65, 82). In the case of the well-characterized IPO9 cargo, the histone H2A–H2B dimer, RanGTP, is capable of binding IPO9–H2A–H2B in a tripartite complex without stimulating cargo release (42, 65, 82). Histone release in the nucleus is thought to be mediated by RanGTP in concert with nucleic acid binding instead. As such, it remains unclear whether actin release from IPO9 can be driven by RanGTP alone or whether additional release factors may be necessary.

**Figure 6:**
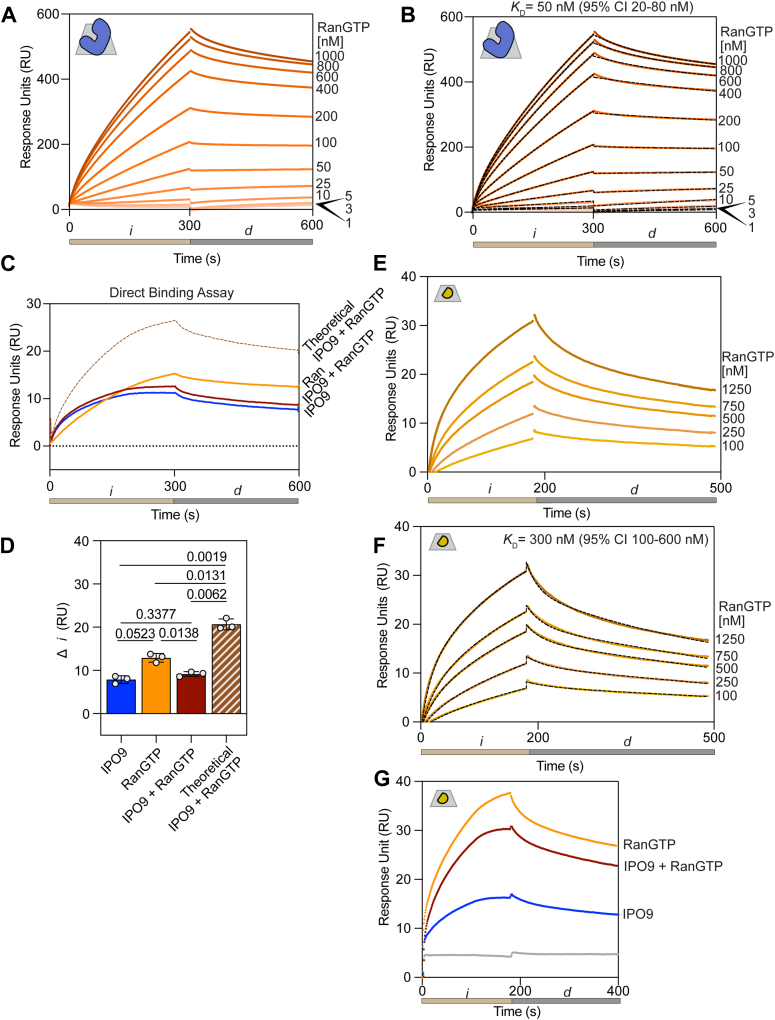
**A tripartite complex between IPO9, RanGTP, and actin does not form**. RanGTP has nanomolar affinity for actin. *A*, representative set of curves for the injection of RanGTP (0.0–1.0 μM) onto IPO9 bound to the SPR chip. An injection (300 s, *i*, *tan*), followed by a 300 s dissociation period (*d*), was conducted for three replicates. *B*, representative two-state kinetic fit series for IPO9–RanGTP interaction data. The arithmetic mean *K*_*D*_ was calculated from kinetic fitting with the standard deviation presented on the graph. *C*, direct binding assay of IPO9 coinjected with RanGTP demonstrates that a tripartite complex between IPO9 (400 nM), RanGTP (50 nM), and actin does not form. The theoretical curve (*dashed brown line*) represents the sum of IPO9 and RanGTP individual signals. *D*, quantification of change in response unit (RU) signal for (*C*) for three replicates with *p* values computed by one-way ANOVA with Tukey’s correction for multiple comparisons. IPO9 and RanGTP do not display significantly different changes from the joint RanGTP and IPO9 condition, though all are significantly different than the theoretical sum of the two individual signals. *E*, affinity of RanGTP for actin. Representative sensorgram for a series of RanGTP concentrations (0.00–1.25 μM) binding to actin immobilized on the chip. Association phase (180 s, *i*) and 300 s dissociation (*d*) phase are indicated below. *F*, kinetic fit of RanGTP series binding to actin. Average calculated kinetic *K*_*D*_ displayed on the graph with standard deviation. *G*, direct binding assay of IPO9 (200 nM) coinjected with RanGTP (1000 nM), demonstrating that with excess RanGTP, the coinjected signal is lower than that of RanGTP alone, indicating RanGTP–IPO9 binding decreases the amount of available protein to bind to actin on the chip. IPO9, importin 9; SPR, surface plasmon resonance.

We first validated that IPO9 bound to RanGTP with the expected affinity under the conditions of our prior direct binding assays. Employing a concentration series of RanGTP (0–1000 nM) on immobilized IPO9, we observe specific IPO9–RanGTP complex formation, approaching saturation around 1000 nM. Applying a two-state kinetic fit model, we find a reproducible *K*_*D*_ in the low nanomolar regime (Fig. 6, *A* and *B*), which is corroborated by prior studies employing isothermal titration calorimetry (42). Notably, this suggests that IPO9 binds RanGTP with tighter affinity than it does actin. We then sought to define RanGTP’s ability to preclude actin–IPO9 binding in a direct binding assay. Upon flowing premixed IPO9 (400 nM) and RanGTP (50 nM) over immobilized actin, we observed reduced signal relative to RanGTP alone but increased signal relative to IPO9 alone (Fig. 6, *C* and *D*). This implied that, unlike the histone H2A–H2B dimer, actin is incapable of forming a stable tripartite complex with both IPO9 and Ran-GTP. Quite unexpectedly, we observe a substantial binding signal in the presence of RanGTP alone. Direct binding between actin and RanGTP has not been previously documented. As such, we probed the nature of RanGTP–actin binding, performing a concentration series of RanGTP (0–1250 nM) over immobilized actin (Fig. 6, *E* and *F*). Employing a two-state binding kinetic fit model, we determine a *K*_*D*_ of 300 nM (95% CI, 100–600 nM), indicating a modest affinity of RanGTP for G-actin, approaching the affinity regime of other well-characterized ABPs (52). Given our measured affinities of IPO9–RanGTP (50 nM), IPO9–actin (60–100 nM), and RanGTP–actin (300 nM), we can reasonably conclude that some loss of signal in the IPO9 + RanGTP direct binding assay is due to IPO9–RanGTP binding. In support of this, we tried another direct binding assay with a higher concentration of RanGTP (1000 nM) and a lower IPO9 concentration (200 nM) and showed that there is decreased signal relative to RanGTP injection alone (Fig. 6*G*). Together, these data indicate a surprising RanGTP–actin interaction that is sensitive to IPO9 binding, and that IPO9 does not form a tripartite complex with actin and RanGTP as other IPO9 cargos have been demonstrated to.

## Discussion

The nuclear import of actin is a crucial and highly dynamic process that regulates the abundance of actin monomer and filament pools in both the cytoplasm and the nucleus (28, 31, 32). Here, we report that IPO9 is capable of directly binding to actin monomers. We further find that the binding of the canonical ABPs, cofilin and profilin, to actin is antagonistic to IPO–actin binding. These results represent a significant departure from the current cofilin-dependent model of nuclear actin import, with implications for how the cytoplasmic pool of actin becomes available for import in the first place (23, 24, 34, 43, 44, 45, 46).

### Modes of competition between IPO9 and cytoplasmic ABPs

The observed competitive binding of actin between IPO9 and ABPs could be due to two nonmutually exclusive mechanisms: direct steric clash between IPO9 and the ABP for overlapping binding surfaces on the actin monomer or negative allostery—wherein an ABP-induced conformational change indirectly perturbs a distal IPO9 binding interface in actin or *vice versa*. While likely to be a composite of these two mechanisms, below we examine the binding surfaces and allosteric effects induced by our panel of ABPs and then present the evidence in favor of contributions for each of these possibilities, drawing from our data and ABP literature.

It is reasonable to expect some component of negative allostery to contribute to the competitive binding energetics, as all the actin binding partners that we examined in competitive assays impart some conformational changes upon engaging actin monomers. The actin monomer structure is composed of four distinct SDs (1) (Fig. 2*B*)1—the barbed face spans the upper parts of SD 1 and 3, respectively, which surround a region known as the target-binding cleft, whereas the pointed face spans the lower surfaces of SD 2 and 4, respectively, which surround the nucleotide-binding cleft. The relative conformations of these domains depend on the actin polymer state, nucleotide-binding state, or engaging a binding partner (83). Profilin, thymosin beta-4, and LatB perturb the nucleotide-binding cleft. Profilin binding to actin is known to induce changes in the spacing between SD2 and SD4, increasing the solvent accessibility of the nucleotide-binding cleft (84). Conversely, binding of the C-terminal alpha helix of thymosin beta-4 is known to “narrow” the region between SD2 and SD4 to produce a relatively closed actin monomer conformation of these SDs (10). The structurally similar latrunculin A and B macrolides occupy the nucleotide-binding cleft between SD2 and SD4, with local conformational changes extending into elements of these SDs—although the global structure is very modestly changed (overall main chain 0.34 Å RMSD) (80). DNase I predominantly engages the pointed face of actin monomers, binding a disordered region known as the D-loop, located in SD2 (85). DNase I binding is known to reorganize SD2, though most of the local conformational changes are buried by the DNase I contact surface. The more distal consequences of DNase I binding are minimal, with slight motions of SD1 relative to the rest of the structure (54, 77, 85). Finally, while there is no direct structure of cofilin–G-actin, cofilin binding to an actin filament induces a tilting of the SD2 D-loop in a manner opposite to that produced by DNase I, thereby disrupting actin–actin contacts within the filament (60, 85). These conformational changes result in documented negative allosteric effects, which influence inter-ABP competition for binding to actin monomers (10, 86).

In considering the range of these local and distal conformational changes with respect to IPO9, the actin surface induced by several of these ABP complex structures could be indirectly refractory. Of the factors that compete with IPO9, profilin induces the largest conformational change that could alter regions of actin distal to its binding site to impair IPO9 binding, whereas the bulk of DNase I conformational changes are buried and less likely to contribute negative allostery. The reduction of actin–IPO9 binding we observe in the presence of profilin, DNase I, and cofilin could be produced through a composite of the changes in the conformation of the D-loop (DNase I and cofilin) and the nucleotide-binding cleft (profilin), rendering the ABP-bound actin an ineffective import substrate. Even considering only these allosteric effects, our results indicate that defined orientations of multiple, spatially distinct actin domains are required for effective IPO9 binding—underlying a mechanism in which IPO9 wraps around the actin monomer, contacting many regions to bind for nuclear import.

Beyond negative allostery, there are several lines of evidence in favor of a surface competition mechanism dominating the energetics in our IPO9 and ABP experiments. The most sterically demanding ABPs employed in our panel (cofilin and DNase I) compete with IPO9 binding, whereas neither a peptide (thymosin beta-4) nor a small molecule (LatB), which buries far less surface area of actin, competes with IPO9. DNase I imparts minimal global changes to actin upon binding—the local SD2 conformational changes are buried by the DNase I interface, and the impact of DNase I binding beyond this SD is extremely small (85). Thus, the competition we observe is far more likely to reflect occlusion of a pointed face surface of SD2 for IPO9 binding than solvent-inaccessible local SD2 conformational changes and or a very subtle movement of SD1. Three of the actin-binding factors we sampled in competitive assays alter the SD2−SD4 orientation. Thymosin beta-4, whose binding surface extends up from the “target-binding cleft” between SD1 and SD3 into the pointed face between SD2 and SD4, and LatB, which has been shown to induce slight movements to residues in SD2 and SD4, are not competitive with IPO9 binding. Yet, profilin, which upon binding to the barbed face (Fig. 4*A*), induces long-range conformational changes to the actin monomer, which “opens” up the nucleotide-binding cleft of actin between SD2 and SD4, is competitive with IPO9 binding. Collectively, these data argue that neither the relative SD2−SD4 conformation nor the nucleotide-binding cleft surface engaged by thymosin beta-4 and LatB markedly contributes to IPO9 binding, suggesting that steric occlusion of the barbed face may be the predominant mechanism of profilin competition.

**Figure 7:**
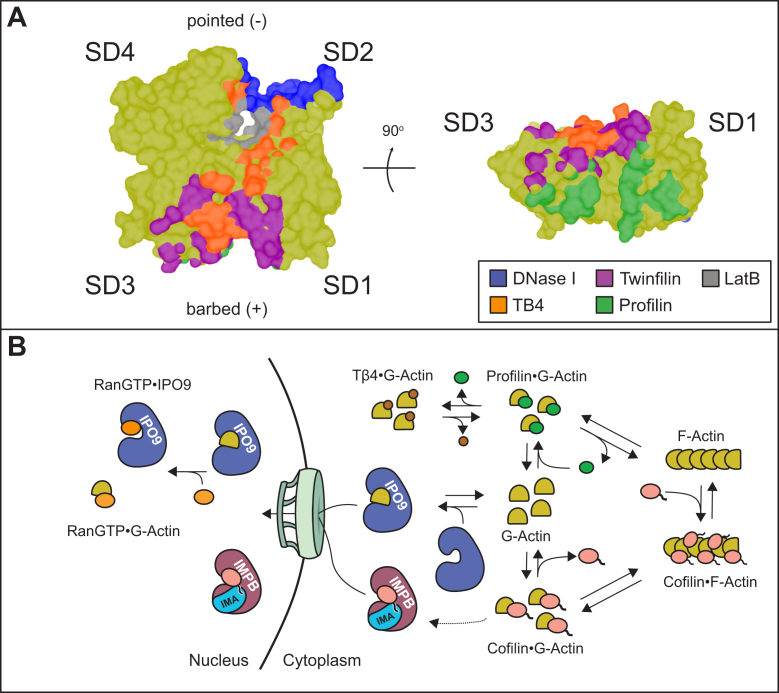
**Revised model of actin nuclear import**. *A*, surface representation of G-actin (*yellow*) overlaid with binding surfaces from DNase I (*blue*, Protein Data Bank [PDB] code: 1ATN), profilin (*green*, PDB code: 2BTF) twinfilin (*purple*, cofilin homolog, PDB code: 3DAW (87)) Latrunculin B (*gray*/*black*, PDB code: 2Q0U) and thymosin beta-4 (*orange*, PDB code: 4PL7). Surface contact landscapes were generated in ChimeraX using a distance of 5 Å from the ABP surface to the actin monomer. Note that some of these surfaces are overlapping, and surfaces are displayed in decreasing priority as follows: latrunculin B, DNase I, thymosin beta-4, profilin, and twinfilin. *B*, revised model of actin import indicating competitive interplay between IPO9 and ABPs for actin monomers in the cytoplasm. Cofilin is critical for filament disassembly and creation of monomers. IPO9 also likely accesses the pool of actin present bound to profilin and thymosin beta-4 in the cytoplasm. RanGTP induces the release of IPO9 from actin once in the nucleus and may have other tertiary interactions with actin. Cofilin can be imported by importin beta (37, 39), so while it is critical for G-actin generation, it does not directly facilitate the import of actin. ABP, actin-binding protein; IPO9, importin 9.

If our competition data is construed to reflect largely competition for shared surfaces, then a more precise model of the actin surface occupied by IPO9 in the import-competent complex emerges (Fig. 7*A*). Finally, IPO9 is a flexible superhelical spiral of HEAT repeats that wraps to envelope cargos (35, 42, 48, 88). Hydroxyl radical footprinting data support the idea that IPO9 predominantly engages cargo by wrapping around it (manuscript in preparation, Keplinger, Ueberheide, and Ruthenburg). Dissociation constants for other cargoes known to be wrapped in their interior cavities are comparable to those we observe for actin (42, 88). As ABPs that engage both barbed and pointed faces of actin on the opposite sides of the molecule compete with IPO9 for actin binding (plausibly through binding overlapping actin surfaces), this is spatially consistent with a wrapping type of IPO9 binding mode. Regardless of the precise mechanisms, our measurements of competition *versus* compatibility define key features of IPO9–actin complex formation, providing a framework for understanding how ABP and IPO9 binding dynamics could impact nuclear import.

### Implications of direct competition between IPO9 and cytoplasmic ABPs for nuclear import

Our finding that IPO9 is competitive with other ABPs emphasizes the dependence of nuclear actin import on the complex equilibria that generate and maintain the cytoplasmic pool of free monomeric actin (Fig. 7*B*). Namely, as cofilin drives the formation of free monomeric actin in its filament-severing activity, and profilin and thymosin beta-4 selectively sequester or release actin monomers, these ABPs are anticipated to become key mediators of the availability of import-competent actin in the cytoplasm (3, 4, 5, 6, 7, 9, 53). In directly competing with several of these ABPs for actin binding, IPO9 becomes sensitive to the availability of free cytoplasmic G-actin, altering the size of the nuclear actin pool in response.

We also note that the concentration of IPO9 in the cytoplasm (0.5 μM) can be orders of magnitude lower than that of other cytoplasmic ABPs (5–500 μM) (48, 49). We posit that the higher direct binding affinity of IPO9 for actin we observe, relative to other actin–ABP interactions, is crucial for its ability to engage a sufficient pool of cytoplasmic monomeric actin for nuclear transport. Actin is distinct from most nuclear-bound cargoes in that its import does not approach complete nuclear localization; rather, the affinity that IPO9 has for actin has been evolutionarily tuned to achieve a delicate balance between cytoplasmic and nuclear pools. Since IPO9 is much less abundant than other ABPs, its actin affinity must be tighter to attain sufficient engagement between proteins. It should also be noted that exportin 6 (XPO6) is constantly functioning in the opposite direction––with both import and export processes reliant on the energy-intensive RanGTP cycle.

Another facet to consider is the competition of monomeric actin with the numerous other IPO9 cargos in the cytoplasm. Cargo binding affinities for IPO9 have been defined in the low-to-mid nanomolar range, such as for the histone H2A–H2B dimer (*K*_*D*_ = 30 nM) and TATA-binding protein (*K*_*D*_ = 1 nM), indicating that a similarly high direct binding affinity will be required for actin to be recognized for nuclear transport (40, 69). Indeed, the lower relative concentration of IPO9 *versus* other ABPs, while having a lower affinity for actin than for other cognate cargo, may ensure that IPO9 does not “overimport” actin into the nucleus but rather maintains a pulse on the abundance of actin in the cytoplasm. Shifting cellular contexts, such as S-phase when histone synthesis and import are massively upregulated, may consequentially affect the import of actin into the nucleus. Similarly, one could imagine that a large increase in cytosolic monomeric actin may impair IPO9-mediated import of other cargos.

In reconsidering the cofilin-dependent model of nuclear actin transport, we note that our measurement of cofilin–actin monomer affinity is considerably weaker than that reported for either profilin–actin or thymosin beta-4–actin (10, 73). As a result, it is unlikely that a pool of monomeric cofilin–actin complexes serves as the main import substrate, given their relatively low abundance. Conversely, while cofilin and actin have numerous mutually dependent functions in the cytoplasm and nucleus, cofilin has key nuclear roles independent of actin and *vice versa*. For example, active cofilin has been implicated in driving the nuclear translocation of p53, contributing to the induction of cell-death pathways in primary neurons (72). Similarly, while cofilin has been shown to associate with some pools of nuclear actin (73), actin’s association with chromatin remodeling complexes has been shown to be dependent on entirely different actin-related proteins (Arps) (74). If the nuclear import of actin and cofilin was uncoupled, as we propose, this would allow for independent regulation of the concentrations of both proteins, in a manner responsive to the respective functions of these proteins in both the nuclear and cytoplasmic compartments. This interpretation is supported by the identification of parallel mechanisms for cofilin’s nuclear import; importin-α/β is known to bind cofilin *via* its bipartite cNLS for import into the nucleus (34, 37).

### Reconciliation of the new model with previous *in cellulo* findings

While our new model of actin’s nuclear transport differs markedly from the extant model, we believe that our results can be reconciled with the prior cell-based findings, when accounting for nuclear import and export as concentration-driven processes. For example, enhanced nuclear localization of both actin and cofilin has been observed upon treatment of live cells with LatB (36). As LatB destabilizes actin filaments, it would drastically increase the concentration of free monomeric actin in the cytoplasm, which in turn would drive the formation of cytosolic IPO9–actin complexes at the expense of other cargos, likely resulting in increased actin import independent of cofilin (62). We note that IPO9 can still bind strongly to actin monomers in the presence of saturating LatB (Fig. 5, *E* and *F*), consistent with robust IPO9-driven actin import following cell treatment with LatB. Simultaneously, this drastic perturbation of filaments would also likely increase the amount of free cofilin, as cofilin preferentially binds filaments over monomers (75); this free cofilin could then be available for import into the nucleus *via* importin-α/β, as shown previously (34, 36, 37). Similarly, we posit that the dependence of nuclear actin transport on cofilin activity observed in knockdown or immunodepletion experiments is not a consequence of the presence of a nuclear actin import complex consisting of actin, IPO9, and cofilin (33). Rather, as cofilin is critical to the generation of a pool of monomeric actin, its depletion in cell extracts results in the decreased availability of an import-competent actin substrate.

### Nuclear RanGTP–actin binding in actin export

Beyond the proposed mechanism for nuclear actin transport that our findings offer, the direct binding we identify between RanGTP and actin has intriguing implications for the mechanism of actin’s nuclear export by XPO6 (31, 32, 35). Before recognizing and binding to profilin–actin for nuclear export, XPO6 must first bind RanGTP to enter a cargo-binding–competent conformation (31). If RanGTP can bind directly to actin, it is possible that this interaction could further stabilize the binding between profilin–actin and the exportin, promoting the assembly or enhancing the structural integrity of the actin export complex. Furthermore, as Ran is loaded with GTP by the chromatin-bound guanosine nucleotide exchange factor RCC1 (89), the affinity of actin for RanGTP could thus have implications for the recruitment and localization of actin on chromatin during transcription, nucleosome remodeling, and DNA damage repair.

### Caveats

It is important to note that these observations are based on *in vitro* experiments; the processes occurring within the cell involve additional layers of complexity, including other binding partners, post-translational modifications, and modes of spatial regulation that we have not examined. Furthermore, we note that the binding alone of a cargo to an importin does not demonstrate import. In addition, while our method of ligand immobilization *via* activated ester crosslinking enables monomeric actin to be presented on the chip in a range of orientations, this does not rule out the presence of steric hindrance or avidity effects contributing to the binding interactions that we observe. Still, it is difficult to probe actin, ABPs, or IPO9–cargo interactions *in cellulo*, as perturbations to any are likely to affect their myriad respective roles, causing off-target or confounding effects. In offering novel insights into the nature of actin–IPO9 complex formation in relation to other ABPs, our findings provide a biochemical perspective, which has thus far been absent from prior investigation, enhancing the mechanistic understanding of actin’s nuclear transport by IPO9.

## Experimental procedures

### Cloning

Human cofilin-1, thymosin beta-4, and profilin expression plasmids were a gift from the Kovar Lab. Human IPO9 was cloned from two codon-optimized, IDT-synthesized, gBlocks into a pET16B expression vector that contains an N-terminal His_10_ tag and a C-terminal GST tag and validated by dye-terminated sequencing. RanQ69L was cloned from a gBlock into a pET16B *Escherichia coli*–inducible expression vector that contains an N-terminal His_10_ tag and a C-terminal CL7 tag (90).

### IPO9 purification

Protein expression constructs were transformed into Rosetta 2 (DE3) pLysS competent cells (EMD Millipore) and grown in 1 l LB cultures containing 25 μg/ml chloramphenicol and 100 μg/ml carbenicillin at 37 °C until an absorbance at 600 nm measured to 0.6. Upon reaching an absorbance at 600 nm, cultures were cooled to 18 °C and then induced with 0.4 mM IPTG, then shaken at 18 °C overnight. Cultures were then harvested by centrifugation in a Thermo Sorvall Lynx 6000 at 4000*g* for 20 min at 4 °C and resuspended in 50 ml of buffer A (20 mM Tri–HCl [pH = 8.0], 150 mM NaCl, and 10% w/v glycerol) supplemented with 2 mM DTT, 1 mM PMSF, and 1250 U of benzonase (Millipore, 71205-3). Cell lysis occurred *via* three passes through the Avestin EmulsiFlex-C3 at 15,000 PSI and then was clarified by centrifugation on the Sorvall Lynx 6000 at 30,000*g* for 20 min at 4 °C. The lysate was filtered through a 0.45 μm filter before being loaded onto an ÄKTA Pure FPLC (Cytiva) *via* 50 ml Superloop.

IPO9 was purified to homogeneity by the sequence of Nickel His-Trap, followed by Glutathione Sepharose 4B (GE Healthcare; 25 ml bed in XJ-50 column), followed by cleavage of the GST tag *via* GST-HRV-3C and dialysis into buffer A. The cleaved protein was then isolated *via* nickel purification on a 1 ml Nickel His-Trap column to remove GST and protease, then dialyzed into 1X HBS–EP + buffer (10 mM Hepes–NaOH pH = 7.6, 150 mM NaCl, 3 mM EDTA, 0.005% Tween-20) or storage buffer (20 mM Tris–HCl [pH 7.5], 200 mM NaCl, and 20% glycerol) and flash frozen for storage at −80 °C. SDS-PAGE gel (10%) was run for 70 min at 150 V and then stained with Coomassie blue to visualize protein species. Bradford reactions were performed to quantify IPO9 concentration.

### Cofilin purification

Recombinant human cofilin was purified using a method modified from (91, 92) as follows. A plasmid containing full-length human cofilin-1 (gift of Dr David Kovar [University of Chicago]) was transformed into Rosetta 2 (DE3) pLysS–competent cells and grown at 37 °C in 1 l Terrific broth cultures containing 25 μg/ml chloramphenicol and 100 μg/ml carbenicillin, until the absorbance at 600 nm measured 0.6. The cultures were then induced with 0.5 mM IPTG by shaking at 37 °C for 4 h. The cells were harvested by centrifugation in a Sorvall Lynx 6000 centrifuge at 4000*g* for 25 min and resuspended in cofilin extraction buffer (20 mM Tris–HCl [pH = 7.5], 500 mM NaCl, 1 mM EDTA, and 10% w/v glycerol), supplemented with 0.10% w/v NP-40, 2 mM DTT, 0.5 mM PMSF, and 1250 U of Benzonase (Millipore; 71205-3). Lysis was accomplished with three passages at 15,000 psi with the Avestin EmulsiFlex-C3. The lysate was then clarified by centrifugation at 30,000*g* at 4 °C in a Sorvall Lynx 6000 centrifuge. The clarified lysate was then purified *via* ammonium sulfate precipitation, discarding the fractions insoluble at 50% saturation and soluble at 70% saturation.

The pellet formed after raising the ammonium sulfate concentration to 70% was resuspended in buffer D (10 mM Tris–HCl [pH = 8.0], 250 mM NaCl, 1 mM EDTA, and 2 mM DTT) and then run over a HiLoad 16/60 Superdex 200 pg (S-200) column. The fractions containing cofilin were then dialyzed into buffer S (10 mM Pipes–NaOH [pH = 6.8], 0.5 mM EDTA, and 10 mM 2-mercaptoethanol) overnight. Following buffer exchange, the sample was flown over a DEAE-sepharose and SP-sepharose column in succession, the DEAE column was removed, and then cofilin was eluted from the S column with a 0 to 1 M NaCl gradient elution. The fractions containing cofilin were dialyzed into 1X HBS–EP + buffer (10 mM Hepes–NaOH, 150 mM NaCl, 3 mM EDTA, and 0.005% Tween-20) for use in SPR. A 12% SDS-PAGE gel was run for 80 min at 150 V and then stained with Coomassie Blue to visualize protein species. Bradford reactions were performed to quantify WT and mutant cofilin concentration.

### Profilin purification

Recombinant human profilin was purified according to the method in (93).

### Thymosin beta-4 purification

Human thymosin beta-4 was expressed in BL21-Codon Plus cells (Agilent Technologies) and purified using the C-terminal IMPACT (Intein Mediated Purification with an Affinity chitin-binding tag) system from NEB. Protein-bound beads were incubated rocking at room temperature with 40 mM DTT for 48 h to induce self-cleavage of the intein-CBD from thymosin beta-4. Thymosin beta-4 was further purified using a Hi-Trap Q-column on the ÄKTA pure chromatography system (Cytiva). Protein concentration was calculated by taking the absorbance at 205 nm using the Shimadzu UV-3600 Plus UV–visible–NIR spectrophotometer and using the extinction coefficient of 128,790 M^-1^ cm^-1^.

### RanGTP purification

Rosetta 2 DE3 expression cells were transformed and grown in 2 l flasks containing 1 l LB and carbenicillin (100 μg/ml) and chloramphenicol (25 μg/ml) until the absorbance at 600 nM measured to be 0.6. Liters were induced with 0.4 μM IPTG and grown overnight at 18 °C. Cultures were then harvested by centrifugation in a Thermo Sorvall Lynx 6000 at 4000*g* for 20 min at 4 °C and resuspended in 50 ml of buffer A (20 mM Tris–HCl [pH = 8.0], 150 mM NaCl, and 10% w/v glycerol) supplemented with 2 mM DTT, 1 mM PMSF, and 1250 U of benzonase (Millipore; 71205-3). Cell lysis occurred *via* three passes through the Avestin EmulsiFlex-C3 at 15,000 PSI and then clarified by centrifugation on the Sorvall Lynx 6000 at 30,000*g* for 20 min at 4 °C. Lysate was filtered through a 0.45 μm filter before being loaded onto an ÄKTA Pure FPLC (Cytiva) *via* 50 ml Superloop.

Ran was purified to homogeneity by the sequence of Nickel His-Trap, followed by overnight cleavage of CL7 tag and then an anion exchange column (Cytiva Q HiTrap 4B XL). The cleaved protein was then isolated over an S-200 sizing column. Ran was loaded with GTP nucleotide *via* incubation in 5 mM MgCl_2_ and 10 mM GTP at 4° for 2 h, after which the concentration of MgCl_2_ was brought up to 20 mM and 10 mM EDTA was added. Protein was then dialyzed into 1X HBS–EP + buffer (10 mM Hepes–NaOH [pH = 7.6], 150 mM NaCl, 3 mM EDTA, and 0.005% Tween-20) or storage buffer (20 mM Tris–HCl [pH 7.5], 200 mM NaCl, and 20% glycerol) and flash frozen for storage at −80 °C. The Bradford assay was performed to determine Ran concentration.

### LatB preparation

LatB was purchased as a powder from EMD Millipore (#428020) and resuspended to 12.5 μM in 1X HBS.

### GST-pulldown

GST-IPO9 was purified as detailed above, but the GST tag was not cleaved with HRV-3C protease. Magnetic glutathione beads were incubated in 5% w/v bovine serum albumin (Dot Scientific) and reducing binding buffer (10 mM Tris–HCl [pH 7.5], 50 mM NaCl, 1 mM CaCl_2_, 1 mM EDTA, and 1 mM DTT) overnight at 4 °C, followed by three 1 ml washes in binding buffer. The preblocked beads (5 μl) were incubated in GST-IPO9 (bead + IPO9), whereas 5 μl of the beads were incubated in an equivalent volume of binding buffer (bead only).

After an hour incubation at 4 °C, excess GST-IPO9 was washed away in three 1 ml washes. Then, purified factors (actin, cofilin) were added at concentrations of 250 nM and 500 nM, respectively, to a final volume of 1 ml. The reactions were incubated with rocking for 2 h at 4 °C. Flowthrough was collected and followed by three 1 ml washes of binding buffer. Samples were eluted in 1X SDS *via* bead boiling, run on SDS-PAGE gel, and stained by Sypro Ruby (Thermo Fisher).

### Pyrene–actin spontaneous assembly assay

Actin was purified from chicken skeletal muscle acetone powder as described (55, 94) and then dialyzed into buffer G (2 mM Tris–HCl [pH 8.0], 0.5 mM DTT, 0.2 mM ATP, and 0.1 mM NaN_3_) prior to use. Actin was labeled on Cys-374 with *N-*(1-pyrenyl)iodoacetamide (“pyrene”) as described (55). The labeled actin was then diluted to 20% in unlabeled G-actin and Ca^2+^–buffer G (0.2 mM CaCl_2_, buffer G), and the protein of interest (IPO9 or cofilin) was dialyzed overnight in HBS–EP + buffer before use in assembly assays. Nanodrop was used to quantify actin concentration, and a 10% SDS-PAGE gel was run at 150 V for 70 min to confirm the purity of the purification.

To prepare the polymerization reactions, the protein of interest was diluted to the appropriate concentration in 18 μl of 10X KMEI (500 mM KCl, 100 mM imidazole [pH 7.0], 10 mM MgCl_2_, 10 mM EGTA [pH 7.0]), 0.1 μl Mg^2+^–buffer G (0.2 mM MgCl_2_, buffer G) and HBS–EP + buffer to a final volume of 145 μl in a black polystyrene 96-well plate. In parallel, actin (20% pyrene labeled) was diluted in 10X magnesium exchange buffer (500 μM MgCl_2_, 2 μM EGTA) to a concentration of 1.5 μM in the same well plate. The protein of interest (121 μl) was then rapidly added to 29 μl of the pyrene–actin using a multichannel pipette.

The plate was immediately transferred to a Safire 2 monochromator plate reader (Tecan) to monitor polymerization over 6 h, with an excitation of 367 nm and an emission of 407 nm. Rows were read at 10-s intervals. All replicates were initiated simultaneously in the same row on a given well plate.

### Pyrene–actin steady-state assembly assay

Reactions were prepared in the same manner as the spontaneous assembly assays (55). Upon addition of the protein of interest to the actin polymerization reactions, the plate was covered with parafilm and aluminum foil and then incubated in the dark for 6 h. Once the polymerization reaction reached the steady state, the plate was transferred to the Safire 2 plate reader (Tecan) to monitor polymerization over 30 min, with an excitation of 367 nm and an emission of 407 nm. Rows were read at 10-s intervals. All replicates were initiated simultaneously in the same row on a given well plate.

### Actin cosedimentation assay

Actin was purified and dialyzed into buffer G as described above (94). Actin cosedimentation assays were then performed using a method modified from (55). Briefly, the actin was diluted to a concentration of 11 μM with the addition of 25 μl 10X KMEI, 25 μl 10X magnesium exchange buffer, and Mg^2+^–buffer G to a final volume of 250 μl. It was then allowed to polymerize at room temperature for 2 h.

The cosedimentation reactions were prepared to a final concentration of 3 μM IPO9 and/or 3 μM F-actin as follows. IPO9 was first diluted in 13 μl of 10X KMEI and Mg^2+^–buffer G, prior to the addition of F-actin (“IPO9 + actin”) or an equivalent volume of Mg^2+^–buffer G, with a final reaction volume of 130 μl. An additional “actin-only” reaction was prepared using an equivalent volume of IPO9 storage buffer *in lieu* of IPO9. The reactions were incubated for 1 h at room temperature, after which the 30 μl “total” sample was collected. The reactions were then resolved by ultracentrifugation at 100,000*g* for 20 min in a Beckman TLA-100 fixed-angle rotor. The supernatant was then removed and adjusted to 1x SDS loading buffer, and the pellet was resuspended in 1x SDS loading buffer. The “total,” “supernatant,” and “pellet” fractions were analyzed on Sypro Ruby–stained 15% SDS-polyacrylamide gels and captured on an Amersham Typhoon imager using 461 nM excitation and 635 nM emission spectra, 50 μM pixel size, and autoPMT setting (approximately 600 V).

### SPR affinity assays

Measurements were made at the University of Chicago Biophysics Core (Research Resource Identifier: SCR_017915). A CM5 chip (Cytiva) was equilibrated to room temperature and then inserted into the BiaCore 8k Surface Plasmon Resonance System (Cytiva). Channels were equilibrated twice with 1X HBS (Teknova 20X HBS-EP + pH 7.6, diluted to 1X with Milli-Q water). Channels 1 and 2 were activated with the simultaneous addition of 1 M *N*-hydroxysuccinimide and 1 M 1-ethyl-3-(3-dimethylaminopropyl) carbodiimide hydrochloride at 10 μl/min for 180 s, followed by 0 nM for channel 1 and 20 nM actin for diluted in 10 mM sodium acetate buffer (pH = 4.0) (Bio-Rad ProteOn) for channel 2 at 10 μl/min for 180 s, followed by a 10 min 1X HBS wash step at 30 μl per minute. Both channels were then quenched with 1 M ethanolamine hydrochloride–NaOH (pH = 8.5) at 10 μl/min for 300 s, and then 1X HBS was flowed through the system overnight to allow for equilibration.

Importin 9 was immobilized as explained above using a concentration of 80 nM.

Actin and IPO9 are the immobilized ligands in all SPR experiments, and IPO9, cofilin, profilin, thymosin beta-4, and LatB are in solution analytes. All experimental data are simultaneously normalized against the channel 1, no protein immobilized control to account for nonspecific analyte binding to the chip surface. Direct binding assays were designed with a constant concentration of immobilized actin (1000 RU) or immobilized IPO9 (6000 RU) and a varied concentration of analyte (95).

For IPO9–actin direct binding, a protein concentration series (0–1500 nM) of IPO9 was flown over the chip for 300 s at a rate of 30 μl/min, followed by a 300 s dissociation phase in 1X HBS, then a 40 s phase of 0.45% v/v phosphoric acid regeneration at 100 μl/min between cycles. Additional 180 s washes in 1X HBS at 10 μl/min were performed between cycles to remove excess phosphoric acid and protein. For the equilibrium fitting, a 1020 s injection at 10 μl/min was performed, followed by a 200 s dissociation phase.

For the IPO9–cofilin or IPO9–profilin series, analyte concentrations (0–55,000 nM) and (0–60,000 nM), respectively, were conducted as described above, with a 300 s injection and 300 s dissociation time at a rate of 20 μl/min, followed by 30 s of 0.45% v/v phosphoric acid regeneration at 100 μl/min. Additional 180 s washes in 1X HBS at 10 μl/min were performed between cycles to remove excess phosphoric acid and protein and re-equilibrate the baseline signal. Cofilin and profilin both are of small molecular weight (18 kDa and 14 kDa, respectively); large saturating concentrations were required in competition assays to get a considerable signal of specific binding. This was enhanced by the basic isoelectric point of both ABPs (∼8.5), which causes the protein to interact nonspecifically with the acidic carboxymethyl-dextran chip surface (96).

DNase I was ordered from Thermo Fisher and resuspended to 100 μM in 1X HBS containing 5 mM CaCl_2_ to minimize protease activity.

The IPO9–actin and RanGTP–IPO9 affinity measurements were conducted in triplicate, whereas the cofilin–IPO9, profilin–IPO9, and RanGTP–actin affinity measurements were performed with singular replicates. Calculated *K*_*D*_s for each replicate were individually fit in Biacore8k software using both two-state pseudo–first-order kinetics and the steady-state affinity model, which is based on total RU change over the course of the injection (*i*) (68). RU were normalized to the start of injection, time = 0.

Graphs and statistical tests were generated using GraphPad Prism (Research Resource Identifier: SCR_002798). ANOVA results underwent Dunnett's or Tukey’s multiple hypothesis testing, and the multiplicity-adjusted *p* values are reported either in the relevant figure or can be found in Supplementary Information Table 1.

### Affinity fitting models

#### Kinetic fitting models

Kinetic fitting models were calculated in the Biacore 8k software using the following parameters:

A two-state fitting model was chosen based on the bimodal nature of the dissociation curve.

Two-state model: A + B = AB = AB∗

#### Model parameters

Two-state model was utilized since there is clearly a bimodal exponential decay for dissociation.

dR_1_/d*t* = k_a1_∗C_A∗_(R_max_ -R_1_ – R_2_)- k_d1_∗R_1_ – k_a2∗_R_1_, + k_d2_∗R_2_

dR_2_/d*t* = k_a2_∗R_1_ – k_d2_∗R_2_

R1 and R2 denote the concentration of AB and AB∗ formed, respectively. C_A_ denotes the analyte concentration.

k_a_1 (association rate constant for analyte binding [M^-1^s^-1^]) - local fitting-initial value 1e5

k_d_1 (dissociation rate constant for the complex [s^-1^]) - local fitting - 1e-2

k_a_2 (forward rate constant for the stabilizing change [s^-1^]) - local fitting - 1e-3.

*R*_max_ (analyte binding capacity of the surface [RU] - local fitting - Ymax

Tc (Flow rate–independent component of the mass transfer constant) - local fitting - 1e8.

RI - (bulk refractive index contribution) - constant-0.

Mass transfer effects are calculated as described in the Biacore application guide “Principles of kinetics and affinity analysis (2020).”

Equilibrium fitting values were calculated through Prism “Specific Binding with Hill Slope” nonlinear regression: Y = B_max_∗X^h^/(*K*_*D*_^h^ + X^h^), where

X = analyte concentration

Y = specific binding signal

B_max_ = maximum binding signal

*K*_*D*_ = analyte signal required to reach 1/2_max_ binding

h = Hill coefficient

### Surface competition direct binding assays

Actin was immobilized as detailed above. For the direct binding competition experiments, saturating cofilin (55 μM) and saturating IPO9 (3 μM) or premixed cofilin and IPO9 were added to the chip simultaneously at 20 μl/min for 300 s, followed by a 300 s dissociation period, a 40 s 0.45% v/v phosphoric acid regeneration cycle at 100 μl/min, and a 180 s wash in 1X HBS at 10 μl/min. RUs were compared with solely adding saturating amounts of one component and normalized to *t* = 0 of the buffer-only control.

Direct binding competition experiments with mutant S119A S120A cofilin (0–55,000 nM) were performed exactly as described above with WT cofilin.

The surface competition modified A–B–A assay was performed using the Biacore8k dual competition assay protocol. Cofilin (55 μM) or buffer only (negative control) was prebound for 300 s at a flow rate of 20 μl/min. Immediately after, IPO9 (0.2 μM) was injected for 300 s at 20 μl/min, followed by a 300 s dissociation period, a 40 s 0.45% v/v phosphoric acid regeneration cycle at 100 μl/min, and a 180 s wash in 1X HBS at 10 μl/min. All samples were normalized so that the 300 s mark (after initial injection [i] and before secondary injection [i’]), RUs are equal to 0. Net change in RU of i’ was calculated *via* subtraction of signal from 590 s to 310 s. Repeated-measures paired one-way ANOVA statistical tests were performed in GraphPad (n = 3).

Thymosin beta-4 was used at 5 or 100 μM, and rofiling was used at 30 μM for their respective direct binding assays with IPO9 (1 μM) or (3 μM), respectively (n = 3) and were performed as described above. For each competition assay, both analytes were present at 3-to 100-fold above their *K*_*D*_ to ensure saturation of actin on the chip. LatB was used at 12.5 μM in the same experimental setup as described directly above (n = 3) with (1 μM) IPO9. DNase I was used at 25 μM with 1 μM IPO9 for surface competition assays in 1X HBS with 5 mM CaCl_2_ to minimize DNase I proteolysis. Three technical replicates of each experiment were performed, barring the 100 μM thymosin beta-4 experiment, of which one replicate was performed.

## Structure Visualization

Structures were visualized using UCSF ChimeraX (97, 98, 99).

## Data availability

Data are contained within the article. Any inquiries regarding data should be directed to the corresponding author, Alexander Ruthenburg (aruthenburg@uchicago.edu).

## Supporting information

This article contains supporting information.

## Conflict of interests

The authors declare that they have no conflicts of interest with the contents of this article.

## References

[1] Dominguez R., Holmes K.C. (2011). Actin structure and function. Annu. Rev. Biophys..

[2] Pollard T.D., Cooper J.A. (2009). Actin, a central player in cell shape and movement. Science.

[3] Andrianantoandro E., Pollard T.D. (2006). Mechanism of actin filament turnover by severing and nucleation at different concentrations of ADF/Cofilin. Mol. Cell.

[4] Pavlov D., Muhlrad A., Cooper J., Wear M., Reisler E. (2007). Actin filament severing by cofilin. J. Mol. Biol..

[5] Wong D.Y., Sept D. (2011). The interaction of cofilin with the actin filament. J. Mol. Biol..

[6] Gutsche-Perelroizen I., Lepault J., Ott A., Carlier M.-F. (1999). Filament assembly from profilin-actin. J. Biol. Chem..

[7] Lu J., Pollard T.D. (2001). Profilin binding to poly-L-proline and actin monomers along with ability to catalyze actin nucleotide exchange is required for viability of fission yeast. Mol. Biol. Cell.

[8] Irobi E., Aguda A.H., Larsson M., Guerin C., Yin H.L., Burtnick L.D. (2004). Structural basis of actin sequestration by thymosin-β4: implications for WH2 proteins. EMBO J..

[9] Weber A., Nachmias V.T., Pennise C.R., Pring M., Safer D. (1992). Interaction of thymosin beta 4 with muscle and platelet actin: implications for actin sequestration in resting platelets. Biochemistry.

[10] Xue B., Leyrat C., Grimes J.M., Robinson R.C. (2014). Structural basis of thymosin-β4/profilin exchange leading to actin filament polymerization. Proc. Natl. Acad. Sci. U. S. A..

[11] Caridi C.P., D’Agostino C., Ryu T., Zapotoczny G., Delabaere L., Li X. (2018). Nuclear F-actin and myosins drive relocalization of heterochromatic breaks. Nature.

[12] Schrank B.R., Aparicio T., Li Y., Chang W., Chait B.T., Gundersen G.G. (2018). Nuclear ARP2/3 drives DNA break clustering for homology-directed repair. Nature.

[13] Viita T., Vartiainen M.K. (2017). From cytoskeleton to gene expression: Actin in the nucleus. Handb. Exp. Pharmacol..

[14] Klages-Mundt N.L., Kumar A., Zhang Y., Kapoor P., Shen X. (2018). The nature of actin-family proteins in chromatin-modifying complexes. Front. Genet..

[15] Wei M., Fan X., Ding M., Li R., Shao S., Hou Y. (2020). Nuclear actin regulates inducible transcription by enhancing RNA polymerase II clustering. Sci. Adv..

[16] Hofmann W.A., Stojiljkovic L., Fuchsova B., Vargas G.M., Mavrommatis E., Philimonenko V. (2004). Actin is part of pre-initiation complexes and is necessary for transcription by RNA polymerase II. Nat. Cell. Biol..

[17] Baarlink C., Wang H., Grosse R. (2013). Nuclear actin network assembly by formins regulates the SRF coactivator MAL. Science.

[18] Philimonenko V.V., Zhao J., Iben S., Dingová H., Kyselá K., Kahle M. (2004). Nuclear actin and myosin I are required for RNA polymerase I transcription. Nat. Cell. Biol..

[19] Kyheröinen S., Prajapati B., Sokolova M., Schmitz M., Viita T., Geyer M. (2024). Actin associates with actively elongating genes and binds directly to the Cdk9 subunit of P-TEFb. J Biol Chem..

[20] Dundr M., Ospina J.K., Sung M.-H., John S., Upender M., Ried T. (2007). Actin-dependent intranuclear repositioning of an active gene locus in vivo. J Cell Biol.

[21] Chuang C.-H., Carpenter A.E., Fuchsova B., Johnson T., de Lanerolle P., Belmont A.S. (2006). Long-range directional movement of an interphase chromosome site. Curr. Biol..

[22] Sen B., Xie Z., Thomas M.D., Pattenden S.G., Howard S., McGrath C. (2024). Nuclear actin structure regulates chromatin accessibility. Nat. Commun..

[23] Ulferts S., Lopes M., Miyamoto K., Grosse R. (2024). Nuclear actin dynamics and functions at a glance. J. Cell. Sci..

[24] Kelpsch D.J., Tootle T.L. (2018). Nuclear actin: from discovery to function. Anat. Rec. (Hoboken).

[25] Sharili A.S., Kenny F.N., Vartiainen M.K., Connelly J.T. (2016). Nuclear actin modulates cell motility via transcriptional regulation of adhesive and cytoskeletal genes. Sci. Rep..

[26] Munsie L.N., Truant R. (2012). The role of the cofilin-actin rod stress response in neurodegenerative diseases uncovers potential new drug targets. Bioarchitecture.

[27] Kwartler C.S., Pedroza A.J., Kaw A., Guan P., Ma S., Duan X. (2023). Nuclear smooth muscle α-actin participates in vascular smooth muscle cell differentiation. Nat. Cardiovasc. Res..

[28] McNeill M.C., Wray J., Sala-Newby G.B., Hindmarch C.C.T., Smith S.A., Ebrahimighaei R. (2020). Nuclear actin regulates cell proliferation and migration via inhibition of SRF and TEAD. Biochim. Biophys. Acta Mol. Cell Res..

[29] Grosse R., Vartiainen M.K. (2013). To be or not to be assembled: progressing into nuclear actin filaments. Nat. Rev. Mol. Cell Biol..

[30] McNeill M.C., Li Mow Chee F., Ebrahimighaei R., Sala-Newby G.B., Newby A.C., Hathway T. (2024). Substrate stiffness promotes vascular smooth muscle cell calcification by reducing the levels of nuclear actin monomers. J. Mol. Cell Cardiol..

[31] Stüven T., Hartmann E., Görlich D. (2003). Exportin 6: a novel nuclear export receptor that is specific for profilin·actin complexes. EMBO J..

[32] Schuh M., Ellenberg J. (2006). Nuclear actin: a lack of export allows formation of filaments. Curr. Biol..

[33] Lappalainen P., Kotila T., Jégou A., Romet-Lemonne G. (2022). Biochemical and mechanical regulation of actin dynamics. Nat. Rev. Mol. Cell Biol..

[34] Dopie J., Skarp K.-P., Kaisa Rajakylä E., Tanhuanpää K., Vartiainen M.K. (2012). Active maintenance of nuclear actin by importin 9 supports transcription. Proc. Natl. Acad. Sci. U. S. A..

[35] Wing C.E., Fung H.Y.J., Chook Y.M. (2022). Karyopherin-mediated nucleocytoplasmic transport. Nat. Rev. Mol. Cell Biol..

[36] Pendleton A., Pope B., Weeds A., Koffer A. (2003). Latrunculin B or ATP depletion induces cofilin-dependent translocation of actin into nuclei of mast cells. J. Biol. Chem..

[37] Iida K., Matsumoto S., Yahara I. (1992). The KKRKK sequence is involved in heat shock-induced nuclear translocation of the 18-kDa actin-binding protein, cofilin. Cell Struct. Funct..

[38] Munsie L.N., Desmond C.R., Truant R. (2012). Cofilin nuclear–cytoplasmic shuttling affects cofilin–actin rod formation during stress. J. Cell. Sci..

[39] Abe H., Nagaoka R., Obinata T. (1993). Cytoplasmic localization and nuclear transport of cofilin in cultured myotubes. Exp. Cell. Res..

[40] Hodges J.L., Leslie J.H., Mosammaparast N., Guo Y., Shabanowitz J., Hunt D.F. (2005). Nuclear import of TFIIB is mediated by Kap114p, a karyopherin with multiple cargo-binding domains. Mol. Biol. Cell..

[41] Pemberton L.F., Rosenblum J.S., Blobel G. (1999). Nuclear import of the TATA-binding protein: mediation by the karyopherin Kap114p and a possible mechanism for intranuclear targeting. J. Cell. Biol..

[42] Padavannil A., Sarkar P., Kim S.J., Cagatay T., Jiou J., Brautigam C.A. (2019). Importin-9 wraps around the H2A-H2B core to act as nuclear importer and histone chaperone. eLife.

[43] Hurst V., Shimada K., Gasser S.M. (2019). Nuclear actin and actin-binding proteins in DNA repair. Trends Cell Biol..

[44] Kloc M., Chanana P., Vaughn N., Uosef A., Kubiak J.Z., Ghobrial R.M. (2021). New insights into cellular functions of nuclear actin. Biology (Basel)..

[45] Wollscheid H.-P., Ulrich H.D. (2023). Chromatin meets the cytoskeleton: the importance of nuclear actin dynamics and associated motors for genome stability. DNA Repair (Amst).

[46] Szabó A., Borkúti P., Kovács Z., Kristó I., Vilmos P. (2025). Recent advances in nuclear actin research. Nucleus.

[47] Pantaloni D., Carlier M.F., Coué M., Lal A.A., Brenner S.L., Korn E.D. (1984). The critical concentration of actin in the presence of ATP increases with the number concentration of filaments and approaches the critical concentration of actin.ADP. J. Biol. Chem..

[48] Chook Y.M., Süel K.E. (2011). Nuclear import by Karyopherin-βs: recognition and inhibition. Biochim. Biophys. Acta.

[49] Watts N.R., Zhuang X., Kaufman J.D., Palmer I.W., Dearborn A.D., Coscia S. (2017). Expression and purification of ZASP subdomains and clinically important isoforms: high-affinity binding to G-Actin. Biochemistry.

[50] Corzo J. (2006). Time, the forgotten dimension of ligand binding teaching. Biochem. Mol. Biol. Educ..

[51] Yang W., Musser S.M. (2006). Nuclear import time and transport efficiency depend on importin β concentration. J. Cell. Biol..

[52] Gonzalez Rodriguez S., Wirshing A.C.E., Goodman A.L., Goode B.L. (2023). Cytosolic concentrations of actin binding proteins and the implications for in vivo F-actin turnover. J. Cell. Biol..

[53] Wiśniewski J.R., Hein M.Y., Cox J., Mann M. (2014). A “Proteomic Ruler” for protein copy number and concentration estimation without Spike-in standards. Mol. Cell Proteomics.

[54] Wang H., Robinson R.C., Burtnick L.D. (2010). The structure of native G-actin. Cytoskeleton.

[55] Zimmermann D., Morganthaler A.N., Kovar D.R., Suarez C., Sanchez-Diaz A., Perez P. (2016). Yeast Cytokinesis: Methods and Protocols.

[56] Suarez C., Carroll R.T., Burke T.A., Christensen J.R., Bestul A.J., Sees J.A. (2015). Profilin regulates F-actin network homeostasis by favoring formin over Arp2/3 complex. Dev. Cell.

[57] Weber A., Pennise C.R., Pring M. (1994). DNase I increases the rate constant of depolymerization at the pointed (-) end of actin filaments. Biochemistry.

[58] Podolski J.L., Steck T.L. (1988). Association of deoxyribonuclease I with the pointed ends of actin filaments in human red blood cell membrane skeletons. J. Biol. Chem..

[59] Arya A., Choubey S., Shekhar S. (2024). Actin filament barbed-end depolymerization by combined action of profilin, cofilin, and twinfilin. PRX Life.

[60] Huehn A.R., Bibeau J.P., Schramm A.C., Cao W., De La Cruz E.M., Sindelar C.V. (2020). Structures of cofilin-induced structural changes reveal local and asymmetric perturbations of actin filaments. Proc. Natl. Acad. Sci. U. S. A..

[61] Nair U.B., Joel P.B., Wan Q., Lowey S., Rould M.A., Trybus K.M. (2008). Crystal structures of monomeric actin bound to cytochalasin D. J. Mol. Biol..

[62] Mannherz H.G., Ballweber E., Galla M., Villard S., Granier C., Steegborn C. (2007). Mapping the ADF/Cofilin binding site on monomeric actin by competitive cross-linking and peptide array: evidence for a second binding site on monomeric actin. J. Mol. Biol..

[63] Blanchoin L., Pollard T.D. (1998). Interaction of actin monomers with *Acanthamoeba*Actophorin (ADF/Cofilin) and profilin. J. Biol. Chem..

[64] Karlsson R. (1994). Real-time competitive kinetic analysis of interactions between low-molecular-weight ligands in solution and surface-immobilized receptors. Anal. Biochem..

[65] Jiou J., Shaffer J.M., Bernades N.E., Yee Joyce Fung H., Dias J.K., Arcy S.D. (2023). Mechanism of RanGTP priming H2A-H2B release from Kap114 in an atypical RanGTP•Kap114•H2A-H2B complex. Proc. Natl. Acad. Sci. U. S. A..

[66] Tanaka K., Takeda S., Mitsuoka K., Oda T., Kimura-Sakiyama C., Maéda Y. (2018). Structural basis for cofilin binding and actin filament disassembly. Nat. Commun..

[67] Hughes R.M., Lawrence D.S. (2014). Optogenetic engineering: light-directed cell motility. Angew Chem. Int. Ed. Engl..

[68] Cytiva (2020). Biacore Insight Evaluation Software User Manual.

[69] Gesellchen F., Zimmermann B., Herberg F.W., Ulrich Nienhaus G. (2005). Protein-Ligand Interactions: Methods and Applications.

[70] Graceffa P., Dominguez R. (2003). Crystal structure of monomeric actin in the ATP state. Structural basis of nucleotide-dependent actin dynamics. J. Biol. Chem..

[71] (1993). The structure of crystalline profilin–β-actin. Nature.

[72] Freeman K.W., Bowman B.R., Zetter B.R. (2011). Regenerative protein thymosin beta-4 is a novel regulator of purinergic signaling. FASEB J..

[73] Goldschmidt-Clermont P.J., Furman M.I., Wachsstock D., Safer D., Nachmias V.T., Pollard T.D. (1992). The control of actin nucleotide exchange by thymosin beta 4 and profilin. A potential regulatory mechanism for actin polymerization in cells. Mol. Biol. Cell.

[74] Forwood J.K., Lange A., Zachariae U., Marfori M., Preast C., Grubmüller H. (2010). Quantitative structural analysis of importin-β flexibility: paradigm for solenoid protein structures. Structure.

[75] Alvisi G., Jans D.A. (2016). Secret life of importin-β; solenoid flexibility as the key to transport through the nuclear pore. Acta Crystallogr. D Struct. Biol..

[76] Kappel C., Zachariae U., Dölker N., Grubmüller H. (2010). An unusual hydrophobic core confers extreme flexibility to HEAT repeat proteins. Biophys. J..

[77] Kabsch W., Mannherz H.G., Suck D., Pai E.F., Holmes K.C. (1990). Atomic structure of the actin: dnase I complex. Nature.

[78] Gibbon B.C., Kovar D.R., Staiger C.J. (1999). Latrunculin B has different effects on pollen germination and tube growth. Plant Cell..

[79] Hitchcock S.E. (1980). Actin deoxyroboncuclease I interaction. Depolymerization and nucleotide exchange. J. Biol. Chem..

[80] Morton W.M., Ayscough K.R., McLaughlin P.J. (2000). Latrunculin alters the actin-monomer subunit interface to prevent polymerization. Nat. Cell. Biol..

[81] Allingham J.S., Miles C.O., Rayment I. (2007). A structural basis for regulation of actin polymerization by pectenotoxins. J. Mol. Biol..

[82] Shaffer J.M., Jiou J., Tripathi K., Olaluwoye O.S., Fung H.Y.J., Chook Y.M. (2023). Molecular basis of RanGTP-activated nucleosome assembly with histones H2A-H2B bound to Importin-9. bioRxiv.

[83] Kudryashov D.S., Grintsevich E.E., Rubenstein P.A., Reisler E. (2010). A nucleotide state-sensing region on actin. J. Biol. Chem..

[84] Porta J.C., Borgstahl G.E.O. (2012). Structural basis for profilin-mediated actin nucleotide exchange. J. Mol. Biol..

[85] Dedova I.V., Dedov V.N., Nosworthy N.J., Hambly B.D., dos Remedios C.G. (2002). Cofilin and DNase I affect the conformation of the small domain of actin. Biophys. J..

[86] Ballweber E., Hannappel E., Huff T., Mannherz H.G. (1997). Mapping the binding site of thymosin beta4 on actin by competition with G-actin binding proteins indicates negative co-operativity between binding sites located on opposite subdomains of actin. Biochem. J..

[87] Paavilainen V.O., Oksanen E., Goldman A., Lappalainen P. (2008). Structure of the actin-depolymerizing factor homology domain in complex with actin. J. Cell. Biol..

[88] Liao C., Shankar S., Pi W., Chang C., Ahmed G.R., Chen W. (2020). Karyopherin Kap114p-mediated trans-repression controls ribosomal gene expression under saline stress. EMBO Rep..

[89] Makde R.D., England J.R., Yennawar H.P., Tan S. (2010). Structure of RCC1 chromatin factor bound to the nucleosome core particle. Nature.

[90] Vassylyeva M.N., Klyuyev S., Vassylyev A.D., Wesson H., Zhang Z., Renfrow M.B. (2017). Efficient, ultra-high-affinity chromatography in a one-step purification of complex proteins. Proc. Natl. Acad. Sci. U. S. A..

[91] Yehl J., Kudryashova E., Reisler E., Kudryashov D., Polenova T. (2017). Structural analysis of human cofilin 2/Filamentous actin assemblies: atomic-resolution insights from magic angle spinning NMR spectroscopy. Sci. Rep.

[92] Yonezawa N., Nishida E., Maekawa S., Sakai H. (1988). Studies on the interaction between actin and cofilin purified by a new method. Biochem. J..

[93] Ezezika O.C., Younger N.S., Lu J., Kaiser D.A., Corbin Z.A., Nolen B.J. (2009). Incompatibility with formin Cdc12p prevents human profilin from substituting for fission yeast profilin: insights from crystal structures of fission yeast profilin. J. Biol. Chem..

[94] Spudich J.A., Watt S. (1971). The regulation of rabbit skeletal muscle contraction: I. Biochemical studies of the interaction of the tropomyosin-troponin complex with actin and the proteolytic fragments of myosin. J. Biol. Chem..

[95] (2017). Handbook of Surface Plasmon Resonance.

[96] Gekko K., Noguchi H. (1975). Potentiometric studies of hydrophobic effect on ion binding of ionic dextran derivatives. Biopolymers.

[97] Meng E.C., Goddard T.D., Pettersen E.F., Couch G.S., Pearson Z.J., Morris J.H. (2023). UCSF ChimeraX: Tools for structure building and analysis. Protein Sci..

[98] Pettersen E.F., Goddard T.D., Huang C.C., Meng E.C., Couch G.S., Croll T.I. (2021). UCSF ChimeraX: Structure visualization for researchers, educators, and developers. Protein Sci..

[99] Goddard T.D., Huang C.C., Meng E.C., Pettersen E.F., Couch G.S., Morris J.H. (2018). UCSF ChimeraX: Meeting modern challenges in visualization and analysis. Protein Sci..

