# Supplementary material for: A revised model of nuclear actin import: Importin 9 competes with cofilin, profilin, and RanGTP for actin binding
Source: J Biol Chem. 2025 Dec 30;302(2):111123. doi: 10.1016/j.jbc.2025.111123 (PMC12860355; doi:10.1016/j.jbc.2025.111123)
Supplement: Supplementary Material [file mmc1.docx]

**Manuscript Title:**

**A revised model of nuclear actin import: Importin 9 competes with cofilin, profilin, and RanGTP for actin binding**

**Author List:**

Amanda J. Keplinger^1†^, Prithi A. Srinivasan^1†^, Sarah M. Christensen^2^, Cristian Suarez^1^, Alexander J. Ruthenburg^1,2 *^

^1^Department of Molecular Genetics and Cell Biology, University of Chicago, Chicago, Illinois, 60637, USA

^2^Department of Biochemistry and Molecular Biophysics, University of Chicago, Chicago, Illinois, 60637, USA

*To whom correspondence should be addressed. Tel: 773-702-1067 Email: [aruthenburg@uchicago.edu](mailto:aruthenburg@uchicago.edu)

† These authors contributed equally to this work

**Materials Included:**

Supplemental Figures S1-7

Supplemental Table S1

**Supplemental Figure 1. (A)** 10% SDS-PAGE gel stained with Sypro Ruby depicting purified factors used in Figure 1—recombinantly produced and purified IPO9 and purified chicken-actin. (IPO9, actin), * corresponds to skipped lane between IPO9 and actin. A representative cofilin preparation is presented in Figure S3A. **(B)** GST pulldown assay with purified factors showing GST-IPO9 binding with actin is not enhanced with addition of cofilin. **(C)** Plots of other two further replicates for IPO9 binding to cofilin with kinetic fits depicted in dashed lines, as described in Figure 1E. **(D)** Signal change (dRU) between 10 and 920s, and 10 and 980s. for each concentration. Displayed on graph are calculated hill fit equilibrium K_D_s for each range of time.  **(E)**  Graph showing the change in RU for each time series, 920-10s, 980-10s and 1010-10s, for each concentration regime of IPO9.

**Supplemental Figure 2. (A)** Graph depicting the average of the 10 maximum values for each condition (0, 0.5, 1.0, 1.5 µM IPO9) of the pyrene actin spontaneous assembly assay (one-sided ANOVA test with Dunnet correction). **(B)** Time to half max (calculated using the average values of the steady state reaction (Figure 2F)) was calculated for each concentration series (one-sided ANOVA test with Dunnet correction).  **(C)** Full 10% SDS-polyacrylamide gels stained by Sypro Ruby for each replicate for actin filament cosedimentation experiments. P = pellet, S = supernatant.

**Supplemental Figure 3. (A**) SDS-PAGE gel of purified WT and actin binding mutant S119A+S120A cofilin. Fractions 17 and 18 for each protein were pooled and dialyzed into 1X HBS for use in experiments. **(B)** Sensorgram plot for cofilin•actin affinity measurement. Binding curves

for each concentration of cofilin from [ 0 – 60 µM] are shades of red with concentration denoted on the right. Change in response units (RUs) is proportional to the analyte protein bound. Tan bar (*i*) corresponds to injection of cofilin and grey bar (*d*) corresponds to dissociation phase where buffer is applied at the same flow rate. **(C)** Kinetic fit (black dashes) overlaid on raw sensorgram curves shown in (B) for each concentration using two-state binding model with local fitting in Biacore8k software. Kinetic fit data for each concentration point in each replicate displayed. Average calculated kinetic fit *K*_D_ shown on graph with standard error reported. **(D)** Additional replicates of direct binding assay (IPO9, WT cofilin) and **(E)** Direct binding assay (IPO9, S119A S120A) experiments described in Figure 3C and 3G, respectively. *i* represents injection period of protein and *d* represents buffer only dissociation period. IPO9 (blue), cofilin (peach), IPO9 with cofilin (pink), mutant cofilin (S119A+S120A) (magenta), and mutant cofilin (S119A S120A) with IPO9 (purple.

**Supplemental Figure 4.** Surface competition A-B-A assay confirms cofilin does not increase IPO9 binding. **(A)** Schematic depicting experimental design for surface competition assays in which cofilin saturates the available binding surface, and then IPO9 is flowed over and the change in binding (*i’*) with or without prebound protein (*i*) to the actin surface is calculated. (IPO9=blue, cofilin=peach, actin=yellow). **(B-D)** Sensorgrams of cofilin [55 µM] and IPO9 [3 µM] dual binding surface competition assay **(E)** Quantification of the change in RU for *i’* (310-590 seconds) for cofilin prebound:IPO9 relative to buffer:IPO9. Tan bars below indicate injection periods (*i, i’*) of respective solutions. RM one-way ANOVA with Tukey adjusted *p* values depicted (n = 3)

**Supplemental Figure 5. (A)** Series of concentrations [0-60,000nM] of profilin binding to actin. **(B)** Replicates of direct binding assay using profilin (green) and IPO9 (blue), joint (teal), theoretical addition of profilin and IPO9 signal (brown) **(C)** Direct binding assay replicates of thymosin and IPO9 experiments described in Figure 4. **(D)** Quantification and analysis of 5µM thymosin beta 4 and 3µM IPO9 competition experiments. RM one-way ANOVA with Tukey correction for multiple comparisons was performed, n=3.

**Supplemental Figure 6. (A)** Replicates of 5µM DNase I + IPO9 [1µM] surface competition experiments. **(B)** Replicates of 50µM DNase I +IPO9 surface competition assays. **(C)** Quantification of surface competition assay between 50uM DNaseI and IPO9 [1µM]. **(D)**  Replicates of Latrunculin B [12.5µM] (LatB) and IPO9 [1µM] surface competition experiments.

**Supplemental Figure 7. (A)** Kinetic fits and curves for RanGTP replicates binding to IPO9 [0.0 – 1.0 µM]. 300 second injection *i*, followed by 300 second dissociation, *d*. **(B)** Replicates of direct binding assay with RanGTP [50 nM] and IPO9 [400 nM]

|  |  | [IPO9 µM] |  |  |  |
| --- | --- | --- | --- | --- | --- |
| **2D** | 0.5 | 1 | 1.5 |  |  |
| 0 | 0.1152 | 0.0284 | 0.0434 |  |  |
| **2E** | 0.5 | 1 | 1.5 |  |  |
| 0 | <.0001 | <.0001 | <.0001 |  |  |
|  |  |  |  |  |  |
| **2J** | IPO9+Actin |  |  |  |  |
| IPO9 | 0.039 |  |  |  |  |
| **2K** | IPO9+Actin |  |  |  |  |
| Actin | 0.3007 |  |  |  |  |
| **2G** | 1.5µM IPO9 |  |  |  |  |
| 0µM IPO9 | 0.9486 |  |  |  |  |
| **4C** | Profilin | IPO9 | Profilin+IPO9 | Theoretical(Profilin+IPO9) | |
| Buffer | 0.008 | 0.0142 | 0.006 | 0.0022 |  |
| Profilin |  | 0.2181 | 0.1027 | 0.012 |  |
| IPO9 |  |  | 0.8886 | <0.0001 |  |
| Profilin+IPO9 | |  |  | <0.0001 |  |
| **5B** | IPO9 | IPO9+DnaseI | |  |  |
| DNaseI | 0.0186 | 0.1337 |  |  |  |
| IPO9 |  | 0.029 |  |  |  |
| **5D** | IPO9 | IPO9+DnaseI | |  |  |
| DNaseI | 0.0024 | 0.0094 |  |  |  |
| IPO9 |  | 0.0004 |  |  |  |
| **5F** | IPO9 | LatB+IPO9 | Theoretical LatB+IPO9 | |  |
| LatB | 0.0208 | 0.0215 | 0.017 |  |  |
| IPO9 |  | 0.0141 | 0.7227 |  |  |
| LatB+IPO9 |  |  | 0.0226 |  |  |
| **6D** | RanGTP | IPO9+RanGTP | Theoretical (IPO9+RanGTP) | | |
| IPO9 | 0.0523 | 0.3377 | 0.0019 |  |  |
| RanGTP |  | 0.0138 | 0.0131 |  |  |
| IPO9+RanGTP | |  | 0.0062 |  |  |

**Table S1.** Table depicting full adjusted p-values. Refer to figure legends for the type of statistical testing for each specific assay.
